# Electrochemical Carbon Dioxide Reduction to Ethylene: From Mechanistic Understanding to Catalyst Surface Engineering

**DOI:** 10.1007/s40820-023-01146-x

**Published:** 2023-07-11

**Authors:** Junpeng Qu, Xianjun Cao, Li Gao, Jiayi Li, Lu Li, Yuhan Xie, Yufei Zhao, Jinqiang Zhang, Minghong Wu, Hao Liu

**Affiliations:** 1 Joint International Laboratory on Environmental and Energy Frontier Materials, School of Environmental and Chemical Engineering, Shanghai University, Shanghai, 200444 People’s Republic of China; 2 Centre for Clean Energy Technology, Faculty of Science, University of Technology Sydney, Broadway, Sydney, NSW 2007 Australia; 3 Department of Electrical and Computer Engineering, University of Toronto, 35 St George Street, Toronto, ON M5S 1A4 Canada

**Keywords:** Key steps in CO_2_RR-ethylene, Preferable reaction pathways, Mechanism understanding, Surface engineering strategies of Cu-based catalysts

## Abstract

Electrochemical carbon dioxide reduction reaction (CO_2_RR) provides a promising way to convert CO_2_ to chemicals. The multicarbon (C_2+_) products, especially ethylene, are of great interest due to their versatile industrial applications. However, selectively reducing CO_2_ to ethylene is still challenging as the additional energy required for the C–C coupling step results in large overpotential and many competing products. Nonetheless, mechanistic understanding of the key steps and preferred reaction pathways/conditions, as well as rational design of novel catalysts for ethylene production have been regarded as promising approaches to achieving the highly efficient and selective CO_2_RR. In this review, we first illustrate the key steps for CO_2_RR to ethylene (*e.g.*, CO_2_ adsorption/activation, formation of *CO intermediate, C–C coupling step), offering mechanistic understanding of CO_2_RR conversion to ethylene. Then the alternative reaction pathways and conditions for the formation of ethylene and competitive products (C_1_ and other C_2+_ products) are investigated, guiding the further design and development of preferred conditions for ethylene generation. Engineering strategies of Cu-based catalysts for CO_2_RR-ethylene are further summarized, and the correlations of reaction mechanism/pathways, engineering strategies and selectivity are elaborated. Finally, major challenges and perspectives in the research area of CO_2_RR are proposed for future development and practical applications.

## Highlights

- Three key processes in carbon dioxide reduction reaction (CO_2_RR) for ethylene generation were discussed, including CO_2_ adsorption/activation, *CO intermediates formation, and C-C coupling.
- The preferable mechanism for ethylene over C_1_ and other C_2_ products reaction pathways.
- Engineering strategies of Cu-based catalysts for CO_2_RR-ethylene.

## Graphical abstract

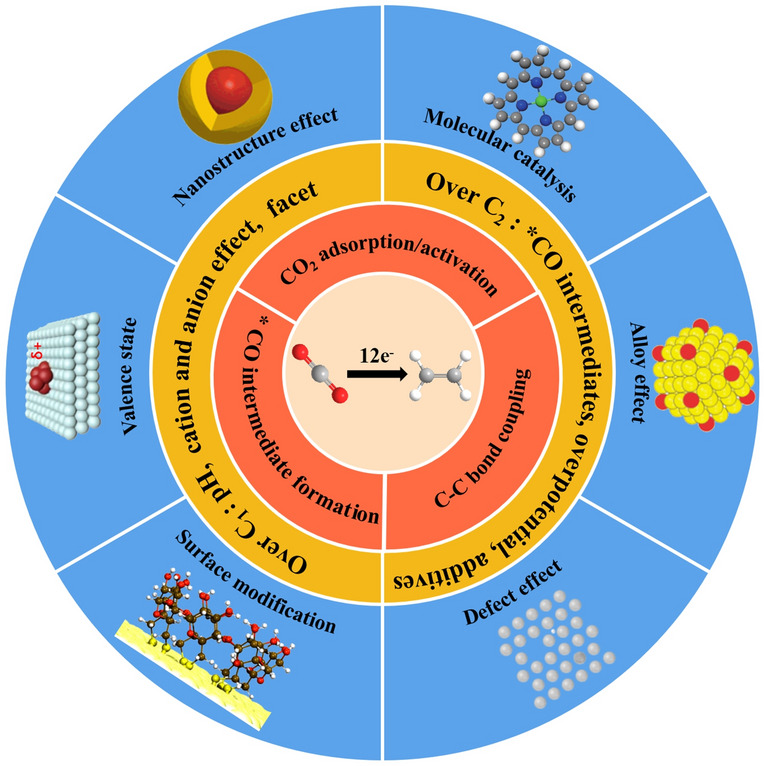

## Introduction

The widespread usage of fossil fuels has destroyed the balance of the carbon cycle, resulting in a sharp increase in carbon dioxide (CO_2_) concentration in the atmosphere, which makes the greenhouse effect an urgent global problem [1–7]. Developing a promising strategy and system to convert CO_2_ into value-added products has attracted intensive research interest. However, CO_2_ is chemically inert and the activation of CO_2_ under ambient conditions is exceptionally challenging [8, 9]. Electrochemical carbon dioxide reduction reaction (CO_2_RR), among the approaches for CO_2_ conversion, is an attractive route that offers a variety of reduction products coupled by multiple proton transfer steps, ranging from C_1_ (e.g., formic acid (HCOOH), carbon monoxide (CO)), C_2_ (e.g., acetic acid (CH_3_COOH), ethylene (C_2_H_4_), ethanol (C_2_H_5_OH,)), C_3_ (e.g., acetone (CH_3_COCH_3_), propanol (C_3_H_7_OH)), and beyond (e.g., n-butane (C_4_H_10_)) [10–13]. In particular, closing the anthropogenic carbon cycle and establishing a sustainable carbon economy are made possible by CO_2_ electrolysis, which is powered by electricity generated from renewable energy sources [14, 15].

Compared to other products, reducing CO_2_ to ethylene is more desirable due to its larger market necessity and has the highest value among all reduction products [16]. For instance, ethylene is the basic raw chemical material for the polymer industry, pharmaceuticals and high-tech material synthesis. In addition, ethylene can be utilized directly as welding fuels or natural gas additives. Currently, ethylene is mainly manufactured by cracking non-renewable naphtha at high temperatures. The production process is energy-consuming and usually has a negative impact on the environment. Conversely, CO_2_RR is an alternative way that is green and sustainable to selectively produce ethylene. Currently, the crucial challenge for converting CO_2_ to ethylene electrochemically is to promote the C–C bond coupling while suppressing the competing reactions such as alternative product formation and hydrogen generation. Cu-based catalysts have been proven to exhibit the best capability for producing ethylene. However, they still suffer from low Faraday efficiency (FE) and poor selectivity. Therefore, it is essential to design novel catalysts for highly efficient CO_2_ conversion to ethylene [17].

The reaction pathways and reactivity of CO_2_RR are heavily reliant on the surface properties and the local reaction environment of catalysts. Slightly alternation of the electrode-electrolyte interfaces may significantly influence the catalytic capability and thus tune the overall catalytic performance [18, 19]. Thus, the sensible design of novel Cu-based catalysts with the geometric effect and electronic effect have been regarded as promising ways to increase the adsorption and desorption energies of the ethylene intermediates, which could lead to high selectivity to ethylene [20–24]. A variety of strategies have been adopted to selectively produce ethylene, including nanostructures control [25–29], defects engineering [30, 31], surface modification [14, 32, 33], and oxidation state alteration [34]. These engineering strategies possess unique mechanisms to promote ethylene production. For instance, altering Cu nanostructures (e.g., constructing unique dendrite structures) can efficiently increase the catalytic active sites and the local pH, benefiting ethylene generation [35]. It is also a common and effective strategy to control the crystal facets and phases to alter the binding energy of intermediates which are also beneficial to the formation of ethylene. Defects can contribute to more active sites, regulate electron distribution, and reduce the energy barrier of C–C coupling. Surface modification by coating molecules (e.g., polytetrafluoroethylene) on Cu surface forms a hydrophobic layer, efficiently restraining the competitive hydrogen evolution reaction (HER) and boosting the C_2_H_4_ yield [36, 37]. Furthermore, Cu^+^ can improve the binding ability of *CO, thus promoting *CO interaction and dimerization [38]. Therefore, engineering Cu-based catalysts to achieve maximized active sites, optimized energy barrier of *CO dimerization, and increased local pH, is essential for selectively producing ethylene.

Although several review articles have summarized Cu-based catalysts for CO_2_RR to produce C_2+_ products, little attention has been drawn to specifically and systematically discussing the engineering effects of novel Cu-based catalysts for ethylene production. In this review, we focus on the key influences on the production of C_2_H_4_, and various engineering strategies on Cu-based catalysts to selectively reduce CO_2_ into C_2_H_4_. We firstly summarize the production mechanisms by reviewing the important steps for CO_2_RR to ethylene, including the CO_2_ adsorption/activation, formation of *CO intermediate, the key C–C coupling step for ethylene formation, to provide an overview understanding of the fundamental principles of CO_2_RR process. Then, we investigate differences in the reaction pathways and conditions for the formation of ethylene and competitive products (C_1_ and other C_2+_), providing a specific guideline to design efficient electrocatalysts. We further discuss the design and engineering strategies of Cu-based catalysts for CO_2_RR conversion to ethylene based on altering the composition, structure, surface state, etc. In particular, the reaction mechanism and correlations between the engineering strategies and selectivity towards ethylene are specifically focused on. Finally, the challenges and perspectives for CO_2_RR are outlined and discussed.

## Fundamental and Mechanism of CO_2_RR to Ethylene

**Fig. 1:**
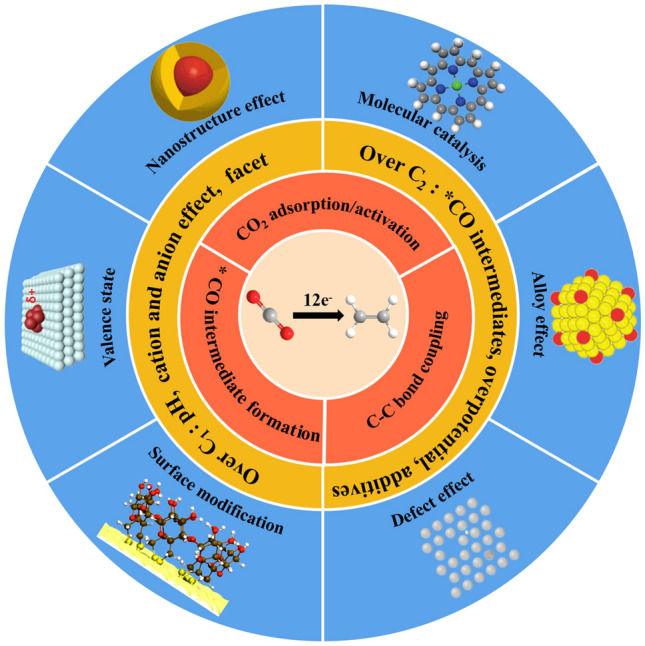
Overview of this review. Three key processes in CO_2_RR for ethylene generation. The preferable mechanism for ethylene over C_1_ and other C_2+_ products reaction pathways. And strategies for designing Cu-based catalysts for CO_2_RR to ethylene

Generally, the electrochemical CO_2_RR occurs at the three-phase interface, including the gaseous reactant CO_2_, liquid electrolyte and solid catalysts. The CO_2_RR process is very complicated due to the presence of many possible intermediates/products with altering the proton-electron transfer, multiple electron transfer and reaction pathways [5]. Specifically, the CO_2_ conversion reaction to ethylene requires a 12-electron transfer with proton coupling, which involves multiple possible intermediates such as *COCHO, *CH*COH, *OCHCH*O and CH_2_CH*O [5, 39–42]. The formation and reaction pathways of these intermediates can be affected by many parameters, such as the electrolyte compositions, applied potentials, temperatures, pH, etc. Therefore, understanding the reaction pathways to selectively generate ethylene is critical for guiding the catalyst design to achieve excellent electrochemical performance. Thus in this section, we will discuss three key processes in CO_2_RR for ethylene generation: CO_2_ adsorption/activation, *CO intermediates formation, and C–C coupling (Fig. 1).

### CO_2_ Adsorption/Activation

**Fig. 2:**
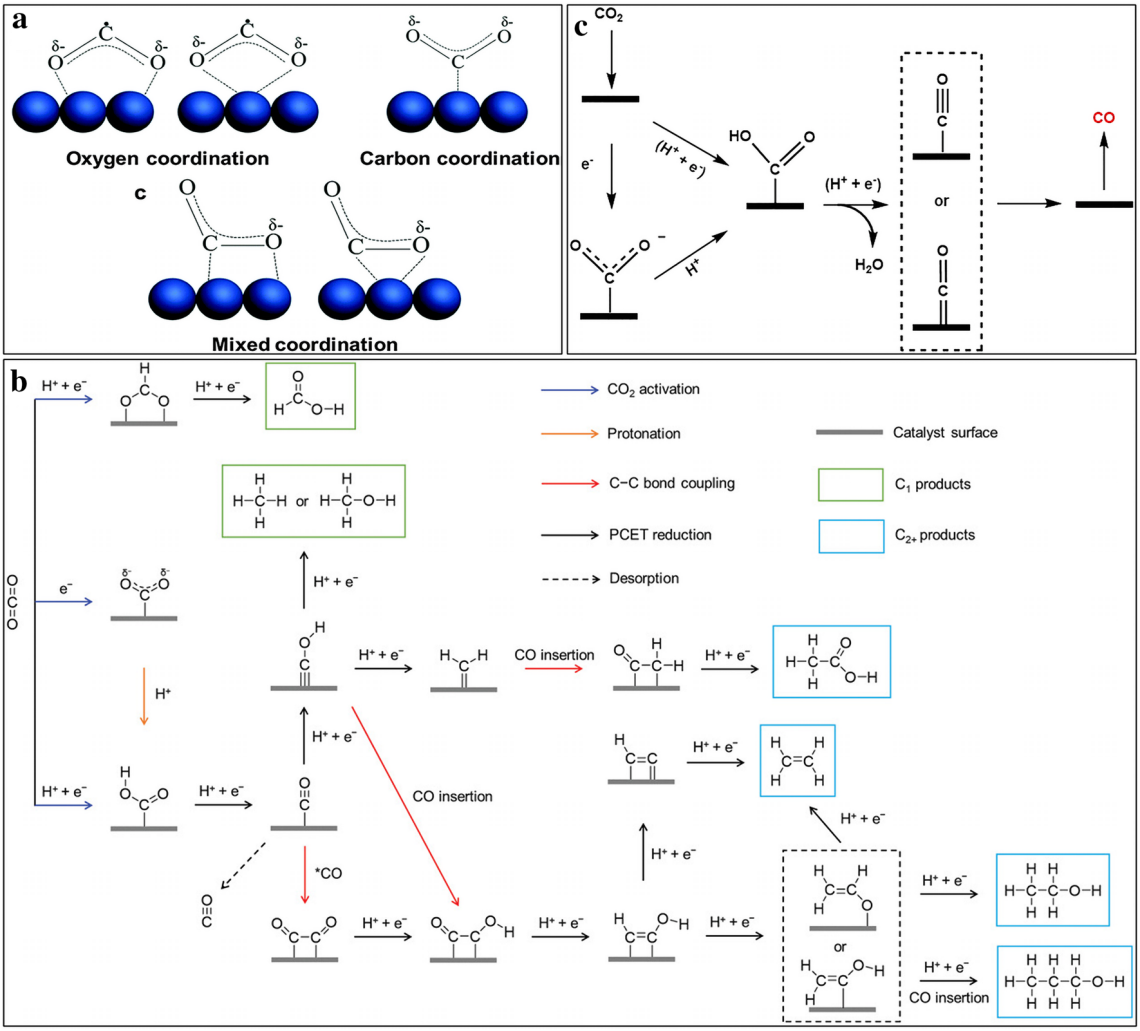
**a** The potential structures of adsorbed CO_2_^δ·−^ on catalysts surface [47]. Reused with approval; Copyright 2008 Royal Society of Chemistry. **b** Potential structure of CO_2_ activation and reaction route of generation to various products. **c** Potential reaction route for electroreduction of CO_2_ to CO [49]. Reused with approval; Copyright 2017 Elsevier

The adsorption and activation of CO_2_ on the active sites in CO_2_RR process are essential for the subsequent reduction process, as optimal energy level can suppress the competing reaction HER [43–46]. Generally, there are two CO_2_ adsorption states on the catalysts. One is to form linear molecules through physical adsorption, and the other is to form charged CO_2_^δ·−^ species through chemical adsorption (Fig. 2a) [45, 47, 48]. A CO_2_^δ·−^ intermediate is usually generated on the catalysts with C atom as the bonding site, when only electrons are involved in the activation process. Alternatively, when both protons and electrons participate in the CO_2_ adsorption/activation process, *COOH is the intermediate with C as binding atoms, followed by the hydroxyl removal and *CO formation, which can be further converted into a number of products, including C_1_ (CH_4_, CH_3_OH, CO) and C_2+_ products (C_2_H_5_OH, C_2_H_4_, CH_3_COOH, C_3_H_7_OH). In contrast, *OCOH intermediates are formed if O atoms act as bonding atoms instead of C, which later are only converted to HCOOH (Fig. 2b) [49].

### *CO Intermediate Formation

The adsorption and activation process generates intermediates that are important for the subsequent reduction reactions. The most important intermediate is *CO which is generated from the as-formed *COOH intermediate by proton coupling electron transfer (PCET) process to remove a molecule of H_2_O. The *CO species can be directly desorbed from the catalysts (e.g., Ag and Au) when *CO adsorption energy is low, forming CO gas (the mechanism of electrochemical CO_2_RR to form CO) (Fig. 2c) [49, 50]. Alternatively, moderate *CO adsorption energy (e.g., Cu) can lead to further coupling and formation of C–C bonds for promoting the following C_2+_ production, based on the Sabatier principle [40]. Furthermore, *CO coverage is also crucial for CO_2_-to-C_2+_, as high coverage of *CO occupies most of the catalytic active sites can not only reduce the hydrogen adsorption and suppress HER, but also increase the chance of C–C coupling, boosting CO_2_ to C_2+_ conversion efficiency [51]. However, it is noteworthy that increasing *CO coverage reduces the affinity of C atoms on Cu surface, which is beneficial to generate oxygenates (especially acetate) rather than ethylene [52]. Therefore, balancing the *CO coverage on the designed catalysts is vital to produce ethylene in CO_2_RR process.

### C–C Bond Coupling

**Fig. 3:**
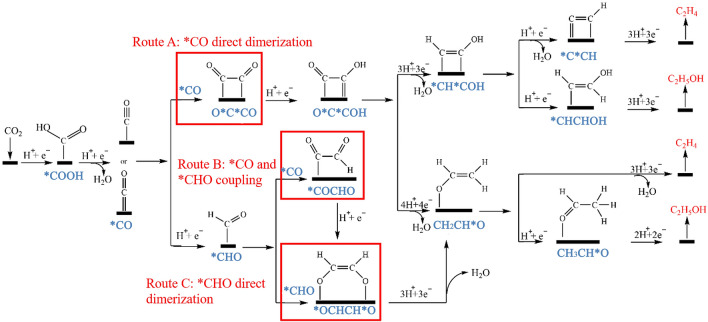
Potential reaction routes for the electroreduction of CO_2_ to ethylene and alcohol [55]. Reused with approval; Copyright 2021 Royal Society of Chemistry

C–C bond coupling is one of the essential steps in the CO_2_RR process to generate C_2+_ products such as ethylene. Three routes for C–C bond coupling have been proposed for the generation of C_2+_ products (Fig. 3).

The first route is the direct dimerization of the adsorbed *CO intermediates resulting in *CO*CO intermediate through C–C coupling [53]. With the aid of proton coupling and electron transfer, the *CO*CO species are further converted into *CO*COH intermediates on Cu(100) [54]. The presence of OH group in *CO*COH intermediates breaks the charge distribution balance from the symmetrical structure of *CO*CO intermediate, which may lead to the appearance of two different reaction intermediates, *CH*COH or CH_2_CH*O, when interactions with protons and electrons. The hydrogenolysis of CH_2_CH*O species produces C_2_H_4_, whereas the hydrogenation of CH_2_CH*O leads to the production of CH_3_CH*O, resulting in the formation of C_2_H_5_OH [55]. The energy barrier of generating C_2_H_4_ by CH_2_CH*O is 0.2 eV lower than that of generating C_2_H_5_OH by CH_3_CH*O [42]. This may be the reason that ethylene is generally more favourable over ethanol on copper-based catalysts. Alternatively, Goddard III and co-workers believed that *CH*COH species is the key intermediate to produce C_2_H_4_ and C_2_H_5_OH, *CH*COH loses OH group to form *C*CH which is then hydrogenated to form C_2_H_4_, whereas, *CH*COH directly hydrogenated to form *CHCHOH which is then hydrogenated to produce C_2_H_5_OH [56].

The second possible reaction route is the direct coupling of *CO intermediates with protons to generate *CHO intermediates, which are preferred on Cu(100) surface at high potential or Cu(111) in the full potential range [40, 57]. The dimerization coupling occurs between *CHO and *CO intermediates to form *COCHO species. Due to the hydrogen electrode model, the adsorption energy of *COCHO is 0.16 eV higher than that of *COCOH in the first route, demonstrating the superior stability of *COCHO intermediates. Therefore, compared with the *COCOH intermediate, *COCHO is a more preferred intermediate by the coupling reaction between *CO and *CHO intermediates [40]. The *COCHO is further hydrogenated to form *OCHCH*O and CH_2_CH*O, which eventually produces ethylene [41, 56, 58].

The third C–C coupling reaction route relies on the dimerization between *CHO intermediates to form the *OCHCH*O species, which is then converted to CH_2_CH*O via H_2_O removal through PCET process, and eventually leads to the ethylene production [39]. It is reported that the dimerization of *CHO intermediate to form C–C bond has more kinetic advantages [59]. For example, the theoretical calculation indicated the energy barrier (0.28 eV) for the formation of C–C bond through *CHO dimerization was much lower than that of the dimerization between *CO and *CHO (1.08 eV) on Cu^0^-Cu^+^atomic interface, indicating that *CHO intermediates were more prone for direct dimerization [60]. In addition, *CO intermediates form C–C bonds directly through dimerization showed a large reaction energy barrier (1.52 eV), thus *CO is preferentially reduced to *COH or *CHO intermediates for further dimerization to produce ethylene [55].

## Alternative Reaction Pathways and Conditions for the Formation of Ethylene and Competitive Products

Although we have established the most preferable theoretical reaction pathways for CO_2_RR to produce ethylene in the previous section, experimentally achieving these reaction pathways remains a great challenge. So far, there have been many factors that can tune the selectivity towards ethylene over the other competing products, such as the microenvironment at the three-phase interface, properties of the catalyst surface and operating conditions, including local pH, cation and anion, surface adsorption substances, catalyst facet, applied overpotential. These physical and/or chemical factors have a vital effect on the thermodynamic adsorption energy of the key intermediates generated in the CO_2_RR process, resulting in the alteration of the reaction pathways [40, 41, 61–70]. In this section, we will discuss the alternative reaction pathways and conditions for the formation of ethylene and competitive products (C_1_ and other C_2+_ products) systematically.

Generally, the morphology of the catalyst surface, hydrophobicity and adsorption of intermediates can lead to the formation of different products. Similarly, the relevant properties of electrolytes, such as pH, CO_2_ solubility and conductivity, can also determine the catalytic performance and selectivity of CO_2_RR, due to their direct contact with the active center. Therefore, in order to tune the reduction product towards ethylene, it is essential to explore the effects of different limiting factors for the alteration of product formation. This section summarizes main aspects based on electrolytes, including the effects of pH, anions, cations and additives, as well as the effects of catalyst surface potential and crystal surface.

### Preferable Mechanism for Ethylene over C_1_ Products Reaction Pathways

The determinative reaction pathway for the generation of ethylene and C_1_ products is the C–C coupling step. The formation of C_1_ products generally comes from the low adsorption energy of the C_1_ intermediates, which cannot stay longer for C–C coupling on the surface catalysts. For instance, the *CO intermediate can be directly desorbed from the catalysts to evolve CO if the adsorption energy of *CO is low. And the *OCOH intermediate generated on the catalysts can be directly reduced to HCOOH through the PCET step. The adsorption energy of intermediates and adsorption sites of intermediates can be altered by many parameters, such as the pH, cation/anion and facet of catalysts, which will significantly influence the reaction pathways to produce ethylene over C_1_ products.

#### ***pH Effect for Ethylene Over C***_***1***_

**Fig. 4:**
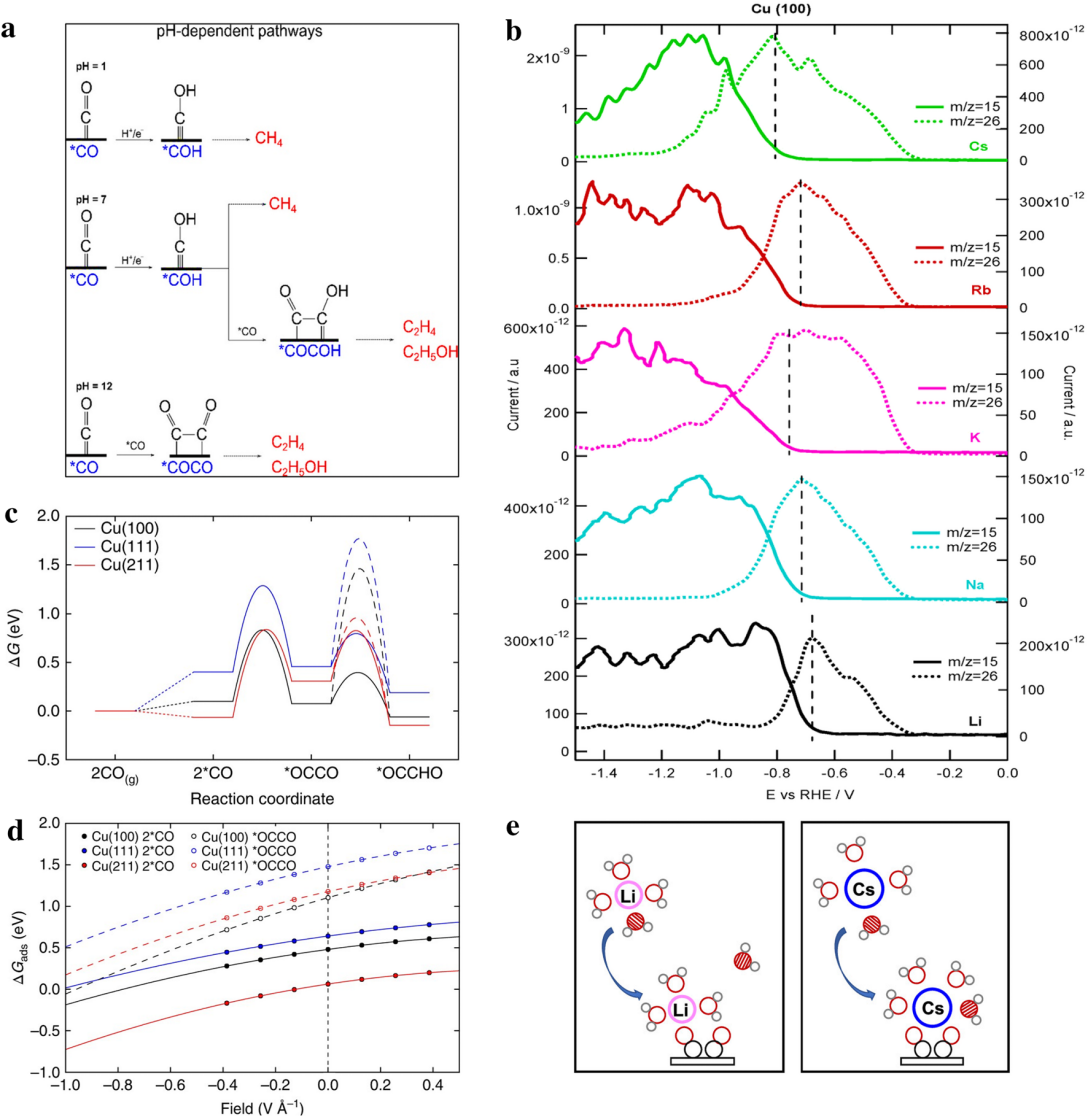
**a** The pH-dependent reaction routes for CH_4_ and C_2_H_4_ on the Cu(111) surface [77]. Reused with approval; Copyright 2016 American Chemical Society. **b** OLEMS signals of CO reduction products in 0.1 M hydroxide solution with different cations. The dotted line corresponds to ethylene, and the solid line corresponds to methane [87]. Reused with approval; Copyright 2017 American Chemical Society. **c** *CO is directly dimerized into the free energy diagram of *CO*CO, the surface hydrogenation is represented by the dotted line and the proton-electron transfer is represented by the solid line form the free energy diagram of *OCCHO, at 0 V vs. RHE. **d** Binding free energy of *CO and *CO*CO on different facets [99]. Reused with approval; Copyright 2018 Springer Nature. **e** Diagram of dynamic coordination between alkali metal ions (Li^+^ and Cs^+^), intermediates and water on CO_2_RR [90]. Reused with approval; Copyright 2021 American Chemical Society

Protons in the electrolyte, especially at the interface between electrode and electrolyte, have a significant effect on the selectivity of reduction products in electrochemical CO_2_RR. When the pH at the interface is low, the C_1_ pathway is the main thermodynamic reaction route [58, 71, 72], e.g., the formation of CH_4_ from the *COH intermediate by *CO hydrogenation [73–76]. At high pH, the dimerization of *C_1_ intermediates (*CO and *COH) is the dominant reaction both thermodynamically and kinetically, whereas the C_1_ pathway is inhibited due to a higher reaction energy barrier, leading to the production of C_2_ products preferably C_2_H_4_ (Fig. 4a). Under neutral conditions, on the other hand, C_1_ intermediates may not proceed through both C_1_ and C_2_ pathways, but could generate multi-carbon products owing to the cross-coupling of these intermediates (e.g., *COH with *CO) [77]. The water molecules binding to the surface of the Cu catalysts play an indispensable role in selectively generating hydrocarbons or oxygenates. Specifically, the surface-adsorbed H_2_O is usually the proton source in the reaction, directly combining *H with the OH group of the adsorbed intermediates, and then removing a molecule of water, thereby promoting the production of hydrocarbons like ethylene. It is worth mentioning that not just the pH of the bulk electrolyte (such as KCl and KHCO_3_) can affect the product distribution. The local pH change at the interface during the CO_2_RR process can also change the selectivity. It has been demonstrated that in electrolytes with weak buffering capacity such as KCl, KClO_4_ and K_2_SO_4_, H^+^ is continuously consumed and the local pH on the electrode surface increases as the reduction reaction progresses, which is favourable for the production of ethylene [78, 79].

Remarkably, the pH value also affects the competitive HER. CO_2_RR and HER occur simultaneously in the aqueous electrolyte, since the redox potential of the proton to H_2_ is very close to the redox potential of CO_2_ conversion, leading to a significant drop of the FE of ethylene [80]. Generally, the production of H_2_ is achieved via the direct reduction of protons in the electrolyte (2H^+^ + 2e^−^ → H_2_) or the reduction of the solvent (2H_2_O + 2e^−^ → H_2_ + 2OH^−^), indicating a low pH is preferred for HER [81]. Therefore, it is necessary to increase the pH value of the electrolyte in order to improve the CO_2_RR while suppressing HER. Furthermore, as high pH is also considered beneficial for C_2+_ production, most studies have been carried out in neutral or alkaline electrolytes [82]. Similarly, designing and controlling the morphology of the electrocatalysts which can increase the local pH value during the reaction is also an excellent strategy to improve the CO_2_ reduction products, such as developing nanowire arrays, dendritic structures, etc. [35, 83]. This will be illustrated in detail in the catalyst design section.

#### Cation and Anion Effect

The change of type or concentration of cations in the electrolyte can significantly affect the activity of CO_2_RR and the selectivity towards the targeted products [78, 84–86]. Cations at the interface can change the microenvironment or interact with the intermediates, which act as promoters in CO_2_RR by changing the free energy pattern and stabilizing specific intermediates. Generally, smaller cations with higher charges are prone to attract more water molecules to form a larger hydration layer, leading to the increase of H^+^. Cations with smaller hydration layers (such as Cs^+^) can get closer to the surface of catalysts and enrich the outer Helmholtz layer, making the interface electric field stronger (Fig. 4b) [87]. The strong dipole interactions between the stronger interface electric field and the surface adsorbate promote the proceeding of CO_2_RR. Meanwhile, solvated cations induced by interfacial electric field can also stabilize CO_2_ and intermediates with different dipole moments through electrostatic interaction. The enrichment of alkali metal cations (such as Cs^+^ and K^+^) in the outer Helmholtz layer can also decrease the activity of competing HER, due to the steric hindrance effect of larger cations to H^+^ on the outer Helmholtz layer [88]. Therefore, larger cations (such as Cs^+^) are more effective in promoting the reaction pathway of the intermediates to selectively produce C_2+_ products, especially ethylene, compared to smaller cations (such as Li^+^) [89]. Furthermore, *CO intermediates have been demonstrated to function as the ligand for cations, which may promote the formation of C–C bond of *CO intermediate, as larger cations have a much higher chance to attract more *CO (Fig. 4e) [90]. Thus, the as-formed cation-intermediate complex is crucial to promote the formation of C_2+_ products. Furthermore, as the radius of alkali metal cations increases from Li^+^, K^+^ to Cs^+^, the free energy barrier of *CO dimerization decreases gradually according to the DFT calculations [91]. The presence of larger cations is also beneficial to the formation of other C_2_ and C_3_ products (such as acetic acid, glycolic acid, ethanol and propanol) owing to the negative potential required for forming *OCCOH from *CO in the presence of Cs^+^ compared to Li^+^ or Na^+^.

In addition to changing the local electric field and stabilizing the reaction intermediate, the hydrated alkali metal cation in the electrolyte can also affect the local pH of the buffer interface [92–94]. The hydrated alkali metal cation will be subjected to the electrostatic interaction of the applied voltage on the cathode, thereby reducing the p*K*_a_ of the hydrolysis [92]. The larger size of the alkali metal cation, the greater the electrostatic effect, leading to smaller p*K*_a_. The hydrolysis reaction is a dynamic equilibrium process [94]. When the p*K*_a_ of the hydrated alkali metal cation is lower than the local pH value of the interface, it will promote the production of H^+^ in the electrolyte buffering the local pH value. This will further alleviate the polarization and Nernst loss caused by the increase of local pH under CO_2_RR conditions and ultimately promote the conversion of CO_2_ instead of H_2_ [95].

The anion effect has been also investigated for CO_2_RR process. Both anions with buffering capacity (bicarbonate (HCO_3_^−^), borate (H_3_BO_3_^−^), phosphate (HPO_4_^2−^)) and anions without buffering capacity (perchlorate (ClO_4_^−^), sulphate (SO_4_^2−^)) have been studied for their influence on the product distribution in CO_2_RR. The results revealed that electrolytes with stronger buffering capacity can contribute protons more effectively, thus are prone to produce H_2_ and CH_4_ while inhibiting the generation of CO, HCOOH, C_2_H_4_ and C_2_H_5_OH, etc. [21, 96, 97]. Therefore, it is preferred to have electrolytes with lower buffer capacity to generate C_2_H_4_ as the main reduction products over C_1_ chemicals and H_2_.

#### ***Facet Effect for Ethylene over C***_***1***_

It has been demonstrated that the selectivity of CO_2_RR process showed a strong dependence on crystal planes of Cu. For example, C_2_H_4_ is prone to be formed on Cu(100), while Cu(111) is favourable for the formation of C_1_ products (CH_4_ and HCOO^-^) [98]. The atomic configuration on the surface of Cu(100) provides the best adsorption geometry for the *CO dimers and stabilises the charged intermediates (*CO*CO) for C_2_H_4_ generation (Fig. 4c, d) [99, 100]. For instance, nanostructured Cu films with more exposed Cu(100) facet could effectively increase the *CO coverage and reduce the C–C coupling energy barrier, achieving FE_C2H4_ of 58.6% at − 0.75 V vs. RHE [46]. However, by adding Cu^2+^ to the electrolyte to passivate the Cu nanosheet (NSs), the dissolution of the Cu NSs was inhibited and the exposed Cu(111) surface was maintained, leading to the selectivity of CH_4_ with 70% FE at − 1.6 V vs. RHE [101]. The instrument characterization results show that Cu(111) surface was prone to produce intermediates such as *COOH and *CH_2_O, which are favourable to forming CH_4_.

### Preferable Mechanism for Ethylene Over Other C_2+_ Products Reaction Pathways

Generally, all C_2+_ products are obtained after C–C coupling step. Therefore, they may share similar reaction intermediates during the reduction processes. Thus, compared to C_1_ vs. ethylene, it is relatively difficult to tune the selectivity between these C_2+_ products. However, it is possible to change the conversion tendency of these shared reactions intermediates to different directions by applying different influencing factors, leading to the formation of different reduction products. For instance, ethylene production can be improved by converting *CH*COH to *C*CH by controlling the content of *CO intermediates. In this section, we mainly discuss the influence of *CO intermediates, overpotential and additives on the reaction pathways of ethylene over other C_2+_ products.

#### *CO Intermediates Effect

**Fig. 5:**
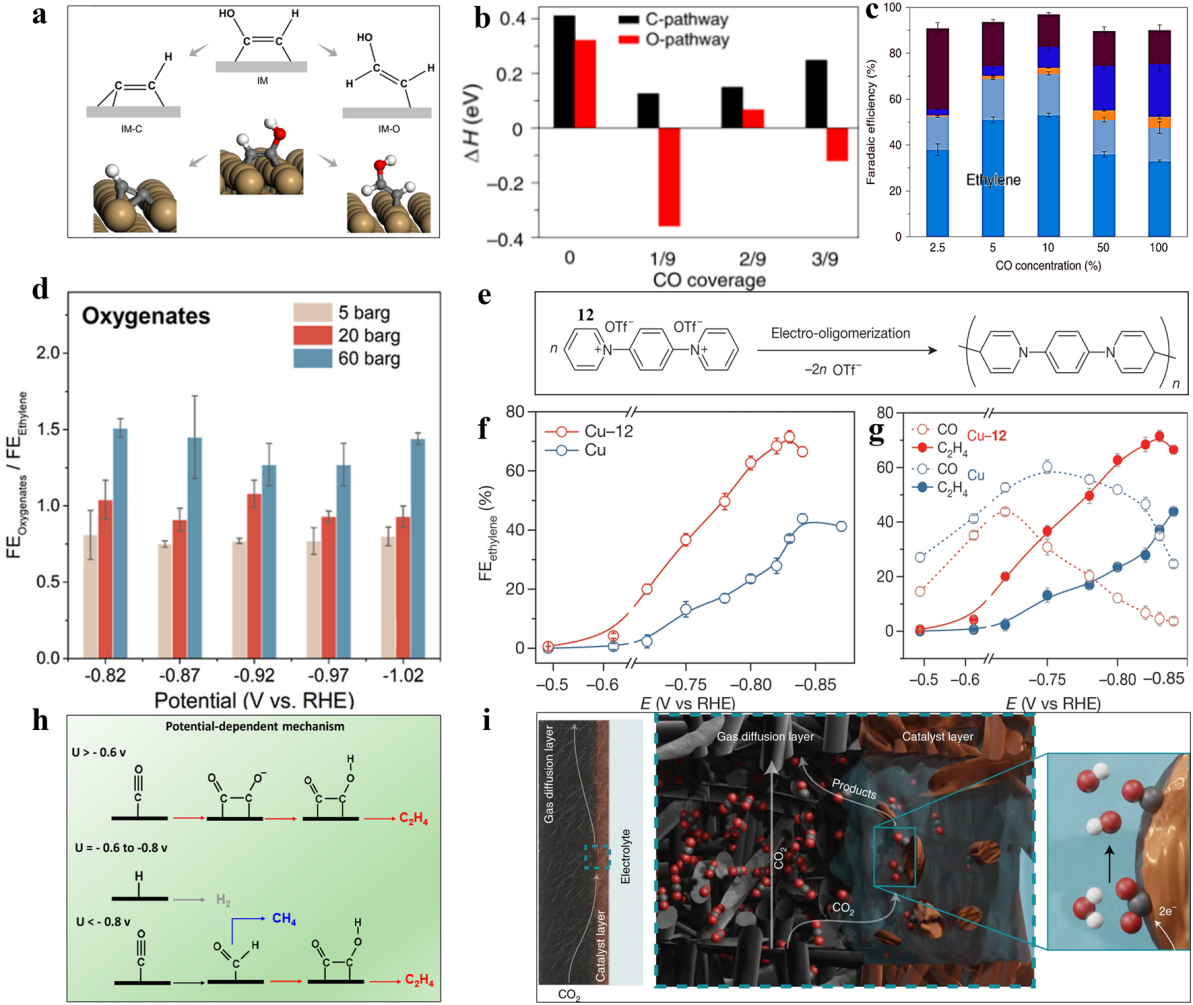
DFT-calculated effects of *CO coverage on selectivity between ethylene and alcohols.** a** A schematic plot of the reaction mechanism where the last oxygen-containing group in *CHCOH (IM) is removed, forming *CCH (IM-C), and an alternative route to *CHCHOH (IM-O), as well as the geometries of IM, IM-C and IM-O on Cu(100) surfaces. **b** Enthalpy changes for the IM-C and IM-O routes at different levels of *CO coverage. **c** FEs of CORR products at various *CO concentrations at − 0.44 V vs. RHE in 1 M KOH [102]. Reused with approval; Copyright 2019 Springer Nature. **d** Pressure-dependent of FE_oxygenates_/FE_ethylene_ at different potentials and pressures on polycrystalline Cu in 0.1 M phosphate buffer with pH = 8 [52]. Reused with approval; Copyright 2022 American Chemical Society. **e** Schematic diagram of N,N’-(1,4-phenylene)bispyridinium salt 12 to form an N-aryl-dihydropyridine-based oligomer. **f** FE of ethylene on Cu and Cu-12. **g** FEs of CO and ethylene on Cu and Cu-12 at − 0.47 V to − 0.84 V vs. RHE [14]. Reused with approval; Copyright 2020 Springer Nature. **h** Potential-dependent reaction routes for CH_4_ and C_2_H_4_ on the Cu(100) surface [98]. Reused with approval; Copyright 2019 American Chemical Society. **i** Surface-interface microenvironment of gas diffusion electrode in CO_2_ electrolysis [104]. Reused with approval; Copyright 2022 Springer Nature

Since *CO intermediates are the keys for C–C coupling to produce C_2+_ products, it is necessary to understand the relationship between *CO intermediates and the formation pathways of ethylene and the competitive products. As shown in Fig. 5a, the *CH*COH (denoted IM) intermediate is the bifurcation point of the ethanol and ethylene reaction pathways [41, 56, 58]. Thus, it is desirable to control the reaction direction from IM to *C*CH (denoted IM-C) while inhibiting the *CHCHOH (denoted IM-O) pathway to promote ethylene production [102]. The theoretical investigation of the reaction energies of IM-C and IM-O pathways unravelled that the lower coverage of *CO on the catalyst’s surface diminished the enthalpy change of the IM-C pathway, benefiting ethylene production rather than ethanol. However, higher *CO coverage is beneficial to the C–C bond coupling and lower free energy barriers of both the IM-C and IM-O pathways, boosting the overall CO_2_RR process to C_2+_ products (Fig. 5b). Therefore, it is vital to maintain proper *CO coverage achieve maximal ethylene selectivity (Fig. 5c) [102]. Intermediates from *CO can also influence the formation of different products. It has shown that *CHO from *CO hydrogenation is a common intermediate for C_2+_ products on Cu. By coupling with another *CO, it can convert to *COCHO, which forms CH*CO through hydrogenation and dehydroxylation [52]. It has been proposed by Abild Pedersen and his colleagues the hydrogenation of CH*CO is the branch point between ethylene and oxygenates. Theoretical calculation results demonstrated that the repulsive interaction between *CO tends to generate oxygenates, due to increasing *CO pressure has to decrease the surface water adsorption, resulting in the low content of protons. Therefore, the increase of *CO coverage is beneficial to the selectivity of oxygenates (especially acetate, requiring a minimum of protons), rather than ethylene (Fig. 5d) [52].

#### Overpotential Effect

The applied overpotential can also influence the CO_2_RR selectivity to produce specific C_2+_ products by altering the stability of intermediates [42, 56]. For instance, *CO*CO prefer to form under low potential, whereas high potential results in the formation of *COCHO (Fig. 5h) [57, 58, 98]. Specifically, on the Cu(100) surface, when the overpotential is higher than − 0.6 V, C–C coupling preferentially proceeds through the *CO*CO pathway, followed by forming *COCHO, leading to the generation of C_2_H_4_ with a lower energy barrier of 0.69 eV. When the overpotential is at the range of − 0.6 to − 0.8 V, *H and *CO share similar adsorption energy, leading to low *CO coverage at the active sites and higher HER. When the overpotential is more negative than − 0.8 V, *COCHO is formed by coupling *CHO with non-adsorbed CO, leading to the formation of C_2_H_4_ and CH_4_ depending on the mass transfer rate of non-adsorbed CO [96, 103]. The theoretical calculations demonstrated that when the overpotential was higher than − 0.6 V, ethylene was the main hydrocarbon product due to the low energy barrier of 0.69 eV for two *CO couples to form a C–C bond [56].

#### ***Additives Effect for Ethylene over C***_***2***_

Incorporating additives in electrolytes affects the microenvironment at the interface of catalysts/electrolytes, which changes the corresponding properties of local electrolytes, such as pH, hydrophobic layer, conductivity, solubility and viscosity. These properties will have a significant effect on the CO_2_RR catalytic performance and selectivity (Fig. 5i) [104–106]. It has been demonstrated that the hydrophilic interface generally promotes the formation of *COOH (then formic acid), while the hydrophobic interface is conducive to the generation of *CO. The surface hydrophobicity property can also limit the proton and water transport, thus forming a locally high pH at the interface, inhibiting the competitive HER [107]. It also possesses the advantage to reduce the thickness of CO_2_ diffusion layer, forming a highly active and stable three-phase interface microenvironment. For example, 1-octadecanethiol was added to the surface of dendritic Cu for catalyst surface modification [32]. Due to the presence of long carbon chains, the contact angle between the Cu surface and the electrolyte increased sharply from 17° to 153°. The improvement of hydrophobicity is more conducive to the capture and release of gas reactants and products at the three-phase interface while inhibiting the competitive reaction (HER). As a result, highly selective production of ethylene was achieved with FE_C2H4_ = 56% at − 1.5 V vs. RHE. Another example is the use of organic molecule (N,Nʹ-(1,4-phenylene)bispyridinium-derived oligomer) to modify the Cu surface (Fig. 5e–g) [14]. The N bader charge in organic molecule affects the production of *CO_atop_, which is more conducive to C–C coupling kinetically. Spectroscopic characterization and simulation calculations showed that the organic molecule adhered to the catalyst surface improved the stability of *CO_atop_, which promote the selectivity to ethylene (FE of 72% at − 0.83V vs. RHE).

## Engineering Strategies of Efficient Catalysts for Electrocatalytic CO_2_RR to Ethylene

So far, the most efficient catalysts in CO_2_RR to produce ethylene are the Cu-based electrocatalysts. However, the pristine Cu catalysts suffer from the insufficient selectivity to ethylene and low overall FE of carbon-based products. There are many strategies proposed to tune the selectivity of Cu catalysts to produce ethylene [108]. For instance, nanoscale Cu catalysts with high porosity and surface areas are beneficial to the microenvironment optimization (e.g., local pH) and reactants/products diffusion [109]. Applying tandem catalyst systems by designing alloys or molecule-Cu catalysts can tune the surface coverage of *CO intermediate, thus optimizing the selectivity for ethylene generation. Moreover, electronic structure tuning is efficient in altering the intrinsic adsorption capability of reactants/intermediates on the catalysts. Heteroatom doping, e.g., alloys/bimetallic catalysts, have been widely investigated to modulate the electronic structure for high ethylene selectivity. Moreover, specific facet exposure may also serve as an efficient approach to regulate the adsorption of intermediates to generate ethylene. Therefore, efficient engineering strategies such as nanostructure control, molecular catalysis, alloy, defects, surface modification, valence state, etc., can be designed for Cu-based catalysts to selectively enhance the ethylene generation rate while inhibiting the competitive products (H_2_, C_1_ and other C_2+_). In this section, we will review various design strategies for Cu-based catalysts, and further investigate the correlation among these strategies, induced specific effects and selectivity for highly efficient producing ethylene.

### Nanostructure Effect

The nanostructure of the designed Cu-based catalysts, including morphology, size, crystal facet, crystal phase, etc., have great influences on the catalytic activity and selectivity of CO_2_RR towards ethylene generation. Changing the morphology of catalysts may alter the local pH or reactants concentration near the electrodes. The porous structure is also favourable for the mass transportation of the reactants/intermediates/products. Tuning the exposed facets of the designed catalysts is efficient in regulating the binding strength of intermediates, thus changing the CO_2_RR catalytic activity and selectivity. The size of electrocatalysts affects the number of coordination sites. Therefore, regulating the nanostructure of electrocatalysts is significant for the CO_2_RR catalytic performance.

#### Morphology Effect

The designed catalysts with specific morphology may directly influence the reaction mechanism and pathways of CO_2_RR, by altering the adsorption energy of intermediates, which results in different selectivity of products [110–114]. For instance, the vertical nanosheet arrays with uniform distribution of Cu catalysts can improve the CO_2_RR efficiency to ethylene by accelerating the mass transfer of CO_2_ in the electrolyte and near the catalyst surface. Dendrites with cavities/tips/needles can efficiently inhibit the competition of HER by increasing pH in the local environment. Cu catalysts with rough surfaces possess larger specific surface areas, increasing the number of active sites for CO_2_ adsorption and activation.

**Fig. 6:**
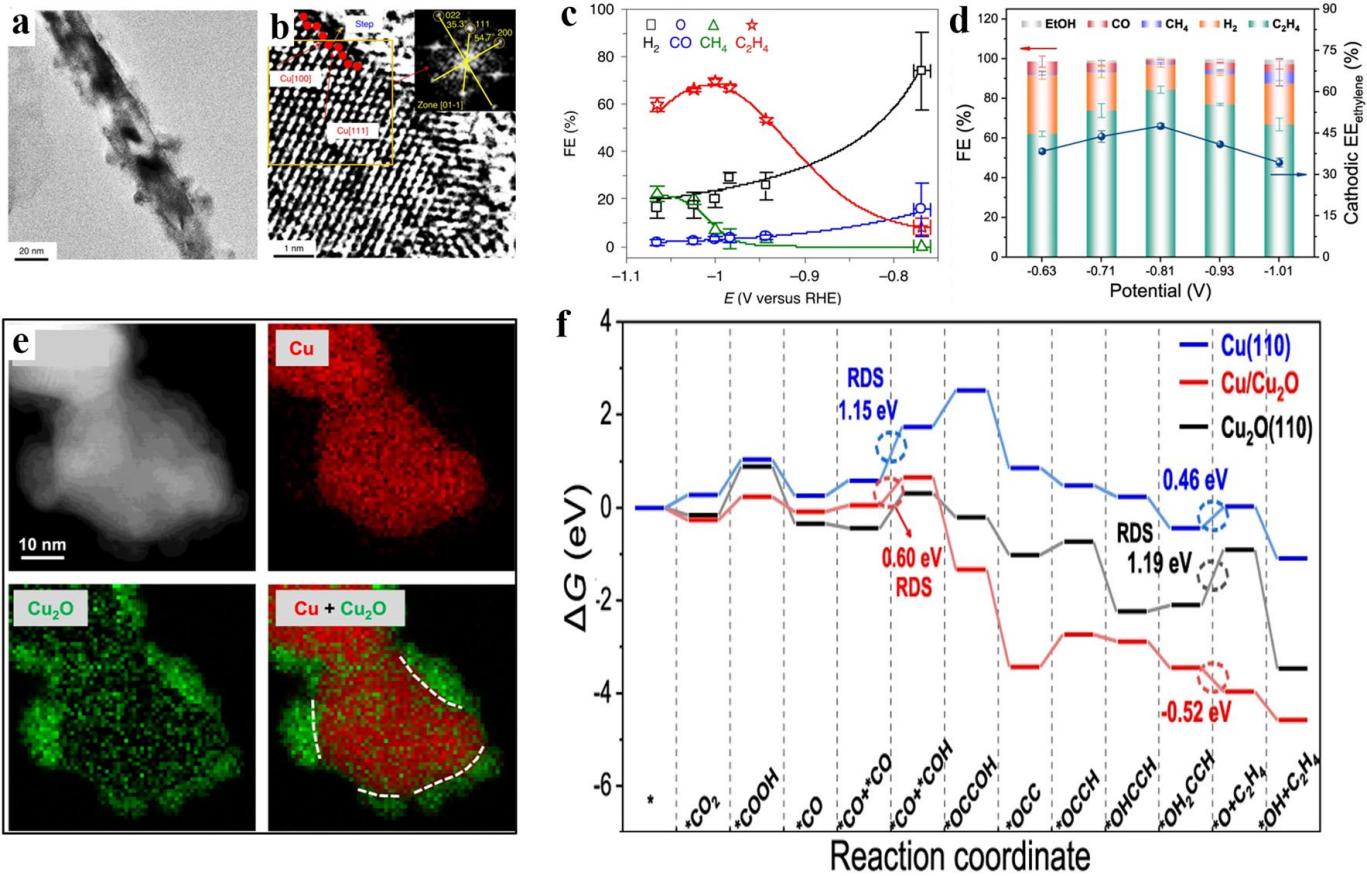
**a** TEM image of A-CuNW. **b** HRTEM image of an A-CuNW with the step structure [27]. Reused with approval; Copyright 2020 Springer Nature. **c** FEs of A-CuNWs at − 0.75 V to − 1.1 V vs. RHE [121]. Reused with approval; Copyright 2018 Elsevier. **d** FEs and the selectivity of C_2_H_4_ of the DVL-Cu@GDL catalyst at − 0.63 V to − 1.01 V vs. RHE in the flow cell. **e** HAADF image and EELS maps in DVL-Cu.** f** Free energy diagram of the CO_2_ to C_2_H_4_ on the Cu_2_O(110) facet, Cu(110) facet and Cu/Cu_2_O interface [122]. Reused with approval; Copyright 2022 Springer Nature

Currently, several morphologies of Cu-based catalysts have been proposed and prepared towards CO_2_RR, including nanoparticles, nanocubes, core-shell structures, nanowires, nanoarrays and nanofoam [83, 115–119]. Specifically, nanoarrays and nanocubes can efficiently promote the selective formation of C_2_H_4_ due to the induced tip effect and changed local pH on the catalyst surface. For example, Cu nanowire arrays have been prepared by electrochemical reduction of CuO nanowire arrays on Cu foil [83]. The derived nanowire arrays with long and dense structures limited the diffusion of the generated OH^−^ at the surface of electrodes [21, 120], which led to the increase of local pH at the interface of electrode/electrolyte. The designed nanoarrays could efficiently inhibit the production of CH_4_ and H_2_, and facilitate the selectivity of C_2+_ products, especially C_2_H_4_. Another example is the Cu nanowires with step surface derived from activating the Cu nanowires at − 1.05 V vs. RHE, which also delivered excellent selectivity towards C_2_H_4_ (Fig. 6a, b) [27]. Theoretical calculation results demonstrated that the adsorption energy barrier of *CO intermediates on two adjacent active sites of the Cu step surface was much lower than that on Cu(100) [121]. The superior thermodynamic stability and lower C–C coupling barrier of the C_2_ path on the step sites led to the maximum FE_C2H4_ = 77.4% at − 1.0 V vs. RHE (Fig. 6c). Other than the nanowire structure, the cubic nanoparticles with branches can also promote the selectivity for C_2_H_4_ generation. For instance, the cubic Cu_2_O nanoparticles with branched CuO (B-CuO) were derived by controlling the oxidation rate in aqueous ammonia via dissolution and reprecipitation mechanism [35]. The as-formed branched CuO nanoparticles induced higher electrochemical active surface area and thus more active sites, which facilitated CO_2_ activation and the subsequent CO_2_RR process. In addition, the accelerated CO_2_RR process resulted in large consumption of protons in the solution and a rapid increase of the interfacial pH, which promoted the C–C coupling and the selectivity to ethylene. Thus, the design of Cu_2_O with the branched CuO nanoparticles delivered a high FE of 70% for C_2_H_4_ at − 1.05 V vs. RHE, which is superior to the original cubic Cu_2_O NPs (FE = 32% at − 1.05 V vs. RHE). Additionally, a 0D-Cu catalyst with dense vertical flake nanostructure (denoted as DVL-Cu) prepared by galvanostatic anodization induced a heterogeneous interface in the CO_2_RR process, which not only effectively avoided agglomeration, but also reduced the C–C dimerization barrier in the C_2_H_4_ formation pathway, boosting the C_2_H_4_ selectivity (Fig. 6d-f) [122]. The Cu catalysts with sharp needle-like morphologies can also promote the C–C coupling and ethylene production due to their capability to nucleate and release small bubbles, leading to an increase in current density and mass transfer [123]. Cu nanoneedles were restored from Cu_2_(OH)_3_Cl through the electro-redeposition method. The total FE_CO2RR_ of this catalyst can reach 75% in 0.1 M KHCO_3_ at − 1.2 V vs. RHE and a high C_2_H_4_/CH_4_ ratio of 200, demonstrating the preference for ethylene products over C_1_ products.

#### Facets Effect

Altering the crystal facets can optimize the catalytic activity and selectivity of the catalysts due to the varied adsorption energy of intermediates on different crystal facets [124–126]. The early research results demonstrated that each facet of Cu may contribute to different product generations. For instance, Cu(100) was beneficial to the formation of C_2_H_4_, Cu(111) was easier to produce C_1_ products, and Cu(110) prefer to C_2_H_5_OH and CH_3_COO^−^ [127–129]. Therefore, reasonable adjustment of the exposed crystal facets can effectively regulate the corresponding catalytic performance of Cu-based catalysts.

**Fig. 7:**
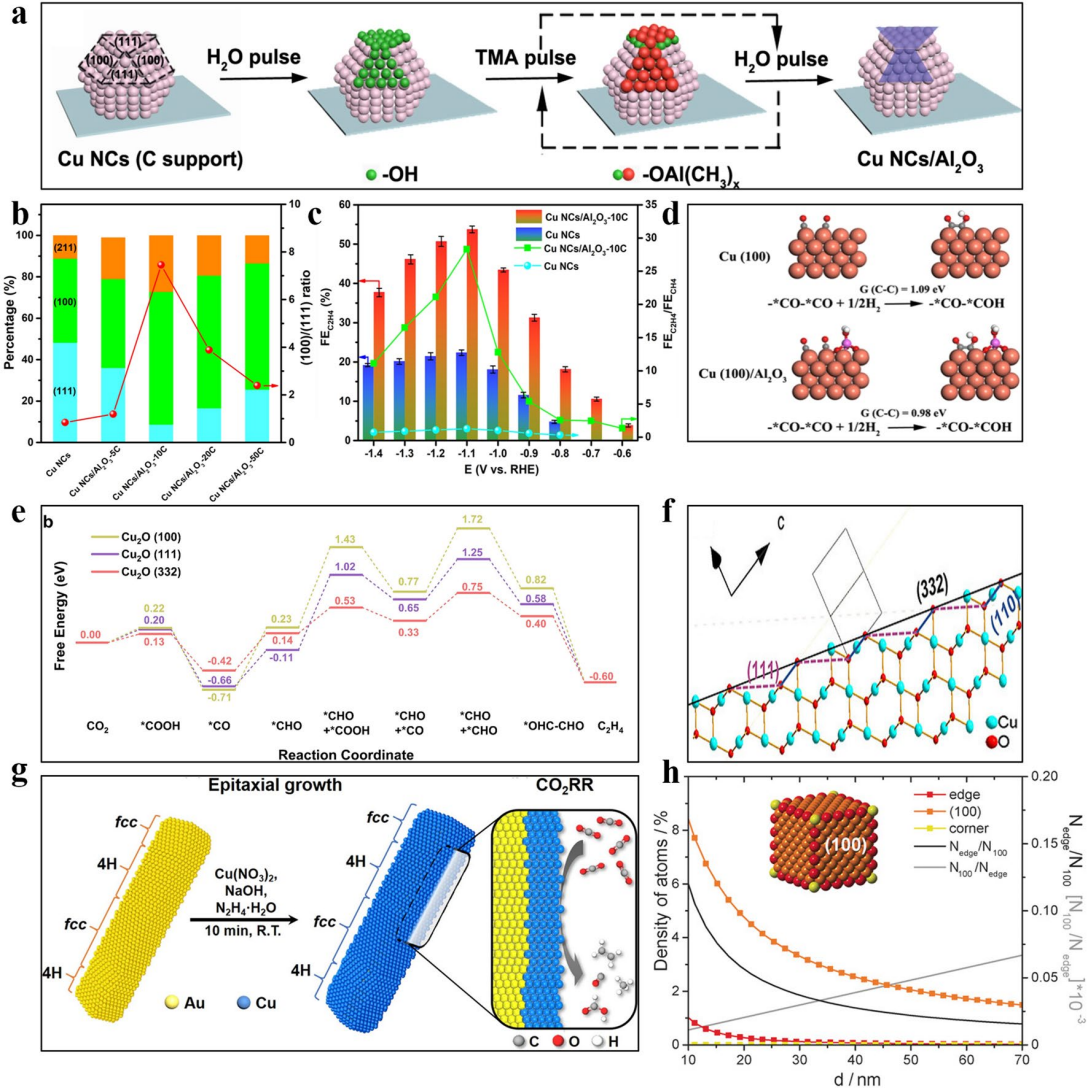
**a** Schematic diagram of preparing FS-ALD with Al_2_O_3_ selectively coated Cu(111) facet. **b** (100)/(111) facet ratio and percentage of Cu nanocrystals with different Al_2_O_3_ coated cycles. **c** FE_C2H4_/FE_CH4_ ratio and FE_C2H4_ of Cu NCs and Cu NCs/Al_2_O_3_-10C at − 0.6 V to − 1.4 V vs. RHE [133]. Reused with approval; Copyright 2021 Wiley. **d **Free energy of dimerized *CO and hydrogenation on Cu(100)/Al_2_O_3_ and Cu(100) [134]. Reused with approval; Copyright 2015 Royal Society of Chemistry. **e** Schematic diagram of the Cu_2_O(332) facet. **f** Gibbs free energy of the reaction intermediates on Cu_2_O(100) (111) and (332) facet [136]. Reused with approval; Copyright 2022 Wiley. **g** Schematic diagram of synthetic 4H/fcc Au@Cu nanorods [28]. Reused with approval; Copyright 2020 American Chemical Society. **h** Relationship between the edge length **d** and the density of the adsorption site and the N_edge_/N_100_ in Cu NC cubes [139]. Reused with approval; Copyright 2014 American Chemical Society

It has been demonstrated that *CO intermediates are prone to be generated on Cu(100) facet compared to other Cu crystal such as Cu(110) and Cu(111), which tends to generate C_2_H_4_. Similarly, *CHO intermediates can also be easily formed on Cu(100), and two *CHO are dimerized to form C_2_H_4_. On the other hand, Cu(111) is more favourable for the formation of *COH intermediates, which generate both CH_4_ and HCOO^−^ [130, 131]. For example, three shaped Cu nanocrystals (NCs) with various facets have been prepared by a colloid method, and the one with Cu(100) delivered high productivity (FE = 57% at − 0.75 V vs. RHE) for C_2_H_4_ [132]. Nanocrystalline Cu with Cu(111) facet, however, has only 12% ethylene selectivity. Thus, increasing the proportion of Cu(100) facet on Cu-based catalysts is an efficient strategy to enhance the selectivity of C_2_H_4_. One example is the Cu foil with a controlled oxidation state and surface morphology derived by using CuCl as a precursor [26]. The stable surface of electropolished Cu(111) is converted to Cu(100) by using HCl to transfer the surface Cu(0) to Cu(I), followed by electrochemical reduction to achieve the reconstruction of Cu surface, leading to high FE for ethylene of 56% at − 2.0 V vs. Ag/AgCl. Moreover, selectively coating Cu(111) to achieve a higher (100)/(111) exposed facet ratio can also enhance the C_2_H_4_ production (Fig. 7b). Ultrathin Al_2_O_3_ has been utilized to be coated on Cu NCs by atomic layer deposition (ALD) technique resulted in a higher FE(C_2_H_4_/CH_4_) ratio of 22 times over pure Cu NCs (Fig. 7a) [133]. The coated catalyst reaches peak performance with a C_2_H_4_ FE of 60.4% at − 1.1 V vs. RHE and 300 mA cm^−2^ in 5 M KOH electrolyte in a gas diffusion electrode (GDE) flow cell (Fig. 7c). The results demonstrated that the presence of Cu(100) facet is critical for the selectivity to C_2_H_4_ in the CO_2_RR process (Fig. 7d) [134]. The issue is that while Cu(100) is more conducive to the formation of C_2_H_4_, Cu(111) is the most stable facet of polycrystalline copper. Therefore, how to expose more Cu(100) and stabilize it in the CO_2_RR process is important and challenging. It is shown that the surface energy of Cu(100) decreases significantly with the increase of the coverage of adsorbed species (including *CO, *COCHO, *COOH etc.) during CO_2_RR, while the surface energy of Cu(100) changes slightly with the increase of the coverage of *H species during HER. The reaction intermediates adsorbed on the surface of Cu acted as capping agents which could benefit the growth of Cu(100). On the contrary, the adsorbed species did not affect the surface energy of Cu(111) either for HER or CO_2_RR. Therefore, due to the lower surface energy of the formed adsorbed species on the Cu(100) facet, it is beneficial to reconstruct the polycrystalline Cu surface into Cu(100) in CO_2_RR condition, while Cu(111) is more easily formed in HER condition [135].

The high-index facet, adding (111) or (110) steps to the (100) surface forming (Cu(711)(7(100) × (111)), can also boost the FE of ethylene [128]. For instance, the star-shaped Cu_2_O nanoparticles with (322)(2(111) × (110)) facets prepared by the reductant-controlled method showed a high FE_C2H4_ value of 74.1% (at − 1.2 V vs. RHE) (Fig. 7e) [136]. DFT calculation revealed that the existence of Cu_2_O(332) surface could significantly reduce the Gibbs free energy of the intermediate process (forming *CHO + *COOH, *CHO + *CO and *CHO + *CHO), thereby significantly improving the selectivity of C_2_H_4_ (Fig. 7f) [39, 137].

In addition to the crystal facet affecting, some non-traditional crystal phases can also effectively regulate the CO_2_RR process. For instance, 4H Au@Cu and heterogeneous 4H/fcc Au@Cu core-shell nanostructures have been prepared by the epitaxial method with the wet chemical method (Fig. 7g) [28]. Both 4H Au@Cu and 4H/fcc Au@Cu delivered high partial current density (*j*_C2H4_) and lower overpotential compared to the conventional face-centered cubic (fcc) Cu, which attributed to the low energy barrier of *CHO intermediates than *COH intermediates on the surface of 4H Cu, inducing preferable pathway for the formation of ethylene.

#### Size Effect

The size of electrocatalysts directly changes the coordination environment of the surface atoms and thereby influences the CO_2_RR catalytic activity and selectivity. Decreasing particle sizes increases surface curvature and lowers the average coordination number of surface atoms [5, 138]. The small size of the Cu catalysts (< 15 nm) may result in the strong adsorption activity of *CO on the low coordinated Cu atoms, leading to the production of H_2_ and CO. With the increase of the size, the proportion of edge sites and corners sites decreases, and thus the generation of H_2_ and CO is suppressed [139]. However, the large size of catalysts may reduce edge active sites, resulting in unsatisfactory catalytic activity (Fig. 7h). Therefore, optimizing the size of Cu catalysts for CO_2_RR is highly favourable to generate desired products. For instance, three Cu cubes with a size of 24, 44 and 63 nm, were prepared by adjusting the reflux temperature and aging time. The Cu cubes with 44 nm have exhibited a maximum FE_C2H4_ of 41% (at − 1.1V vs. RHE) in 0.1 M KHCO_3_ originating from the ideal ratio of plane sites on Cu(100) to edge sites [140]. The size effect can also influence the unsaturated edge sites, which is also significant to the CO_2_RR selectivity, resulting in optimized binding strength of the reaction intermediates [100]. For example, Cu nanoparticles with varied sizes were derived by electro-reducing the pre-prepared Cu_2_O film [141]. The experimental results showed that FE_C2H4_ was inversely proportional to the size of the catalysts, in which the size decreased from 41 to 18 nm, while the FE of C_2_H_4_ increased from 10% to 43%. The smaller Cu particles with higher selectivity for C_2_H_4_ were attributed to more grain boundaries and defects, which significantly enhanced the *CO adsorption and thus the C–C coupling step. Although the above two examples showed contradictory conclusions regarding the size of the Cu nanoparticles, the induced effect by altering the catalyst’s size such as increasing crystal facet (former), active sites (latter), and stronger *CO adsorption energy (latter) is more important for promoting ethylene production.

### Molecular Catalysis

**Fig. 8:**
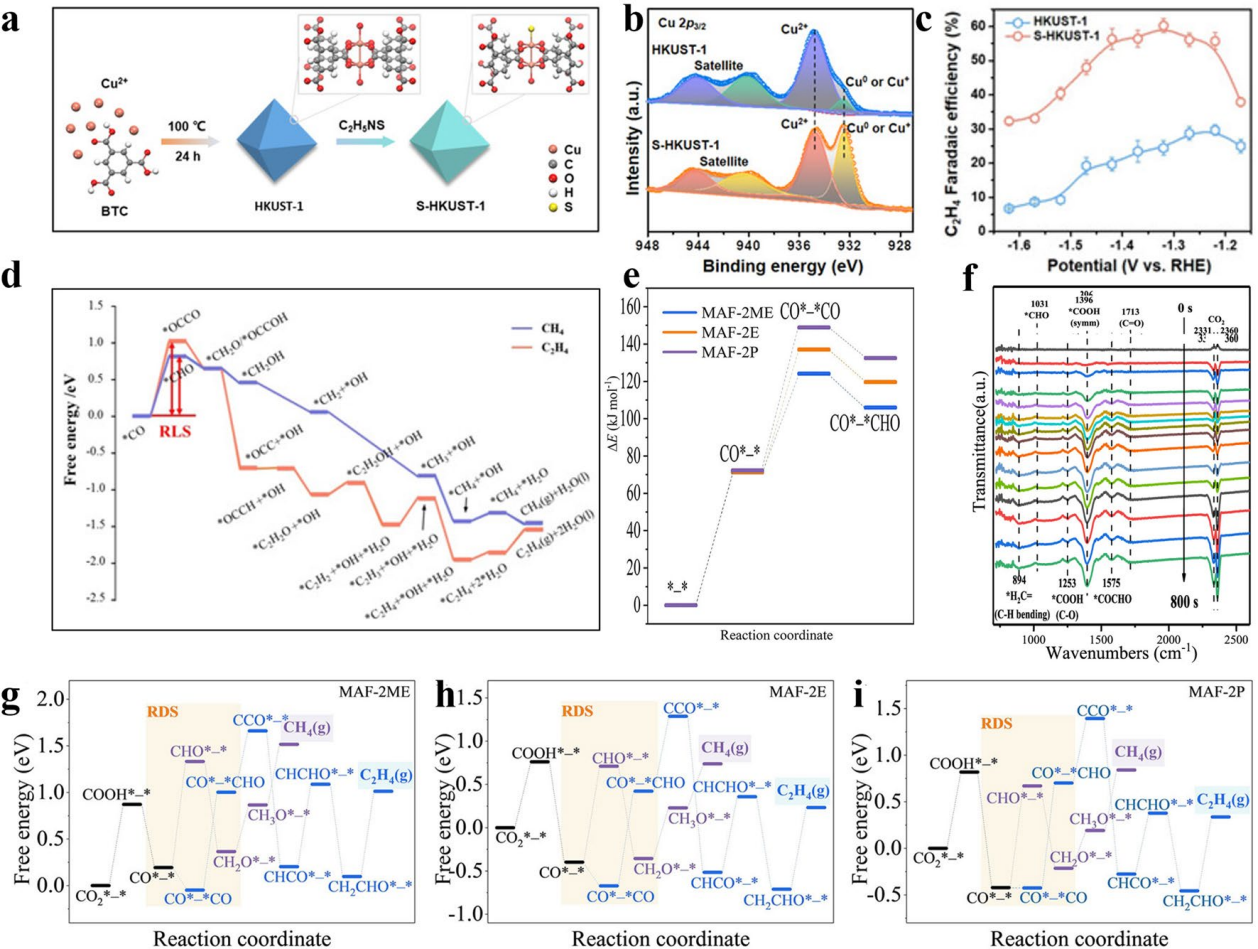
**a** Schematic diagram of preparation of S-HKUST-1 doped with S atom. **b** XPS spectra of S-HKUST-1 catalysts in the Cu_*2p*_ region. **c** FE_C2H4_ of HKUST-1 and S-HKUST-1 at − 1.15 to − 1.6 V vs. RHE. **d** Free energy illustrations for obtaining C_2_H_4_ and CH_4_ on Cu(111) facet [146]. Reused with approval; Copyright 2022 Wiley. **e** In-situ ATR-FTIR spectra of PcCu-Cu-O during the process of electroreduction of CO_2_ to ethylene [147]. Reused with approval; Copyright 2021 American Chemical Society. **f** Framework energies of the catalysts at different CO_2_RR states. The free energy graphic of **g** MAF-2ME,** h** MAF-2E and **i** MAF-2P in proper order [148]. Reused with approval; Copyright 2022 Wiley

Molecules can change the coordination environment of Cu atoms in the catalysts, and the interaction between catalysts and ligands can stabilize the key intermediates. It has been widely studied that modifying Cu by organic macromolecules can form complexes that may be beneficial for ethylene production [142, 143]. For instance, Cu porphyrin (PorCu) and Cu phthalocyanine (CuPc) composed of Cu^+^ and macrocyclic complexes have been investigated to selectively reduce CO_2_ to ethylene [144]. PorCu and CuPC have a tuneable molecular structure and a larger conjugated system, which makes it easier to adjust the electronic structure of center Cu^+^. Moreover, ProCu has exhibited high selectivity and activity to C_2_H_4_, attributing to the OH group in the porphyrin structure as binding sites to the reaction intermediates. It can also provide intramolecular sources of protons, with an oxidation state of Cu^+^ at the center as the active sites [145]. Another example is the surface of the Cu cluster engineered by controlling the paddle-wheel structure of the Cu dimer in the deformed HKUST-1 (C_18_H_6_Cu_3_O_12_, Cu_3_(BTC)_2_·xH_2_O, BTC = benzene-1,3,5-tricarboxylate) MOF [33]. It was found that the Cu clusters with the asymmetric structure were formed from the deformed Cu dimers by heat treatment, resulting in more uncoordinated Cu sites, which were able to increase the FE of C_2_H_4_ from 10% to 45% while reducing the H_2_ yield to less than 7%. Recently, a local sulphur doping strategy has been used to rationally construct isolated Cu-S (S-HKUST-1) on HKUST-1 precatalyst (Fig. 8a) [146]. The catalyst was reconfigured in situ to obtain a two-phase copper/copper sulphide (Cu/Cu_x_S_y_) interface with abundant Cu^δ+^ sites (Fig. 8b). The moderate coupling site distance of stable Cu^δ+^ reduced the kinetic barrier for the dimerization of the *CO intermediates, demonstrating a high C_2_H_4_ selectivity up to 57.2% at − 1.32 V vs. RHE at the operated a *j* of 400 mA cm^−2^ (Fig. 8c, d). Moreover, dual active sites with PcCu-(OH)_8_ as ligands and square planar CuO_4_ as nodes (PcCu-Cu-O) have been investigated for highly efficient CO_2_RR [147]. The results demonstrated that the derived PcCu-Cu-O exhibited up to 50% FE_C2H4_ and a high current density of 7.3 mA cm^-2^ at − 1.2 V vs. RHE. The excellent catalytic activity and selectivity are attributed to the synergistic effect between PcCu (*CHO formation sites) and CuO_4_ (*CO formation sites), reducing the energy barrier of C–C coupling step (easier than the direct coupling of *CO intermediates), thus boosting the ethylene selectivity in CO_2_RR process (Fig. 8e). Another flexible and tunable Cu(I) triazole framework was also developed as a novel MOFs material for electrocatalytic CO_2_ reduction, in which the Cu_2_O and dialkyl-1,2,4-triazoles were reacted to produce isoreticular analogs of MAF-2 ([Cu(detz)], Hdetz = 3,5-diethyl-1,2,4-triazole) [148]. By changing the size of the non-coordinating ligand side groups, MAF-2E (i.e., MAF-2, side group are two ethyl groups) was derived, resulting in high FE_C2H4_ up to 51.2% (− 1.30 V vs. RHE) and excellent stability over 10 h. Computational simulations showed that compared with MAF-2P (side group are two propyl groups), although the binuclear copper sites in the structure could allow the intermediate to have C–C coupling, the smaller ethyl groups had less spatial steric resistance to binuclear Cu sites, which are more favourable for the C–C coupling process to promote the C_2_H_4_ generation (Fig. 8f–i). Moreover, the side groups of two ethyl groups increased the hydrophobicity of the material and inhibited the competition of HER.

### Alloy Effect

By combining two or more complementary metals into heterogeneous catalysts, the selectivity, activity and stability of these catalysts can be further improved for CO_2_RR due to the altered electronic structure and geometric effects [5, 45, 60]. Recently, alloying different metal species with Cu to form bimetallic catalysts has been widely investigated, which showed a significant change in selectivity. For instance, CuIn and CuAu are reported to improve the FE of CO, while CuSn and CuPd can promote the production of HCOO^-^ [60]. CuAg and CuZn, on the other hand, are slightly different. The main products for CO_2_RR using these two catalyst species can be easily altered by changing the metal ratios and preparation methods.

**Fig. 9:**
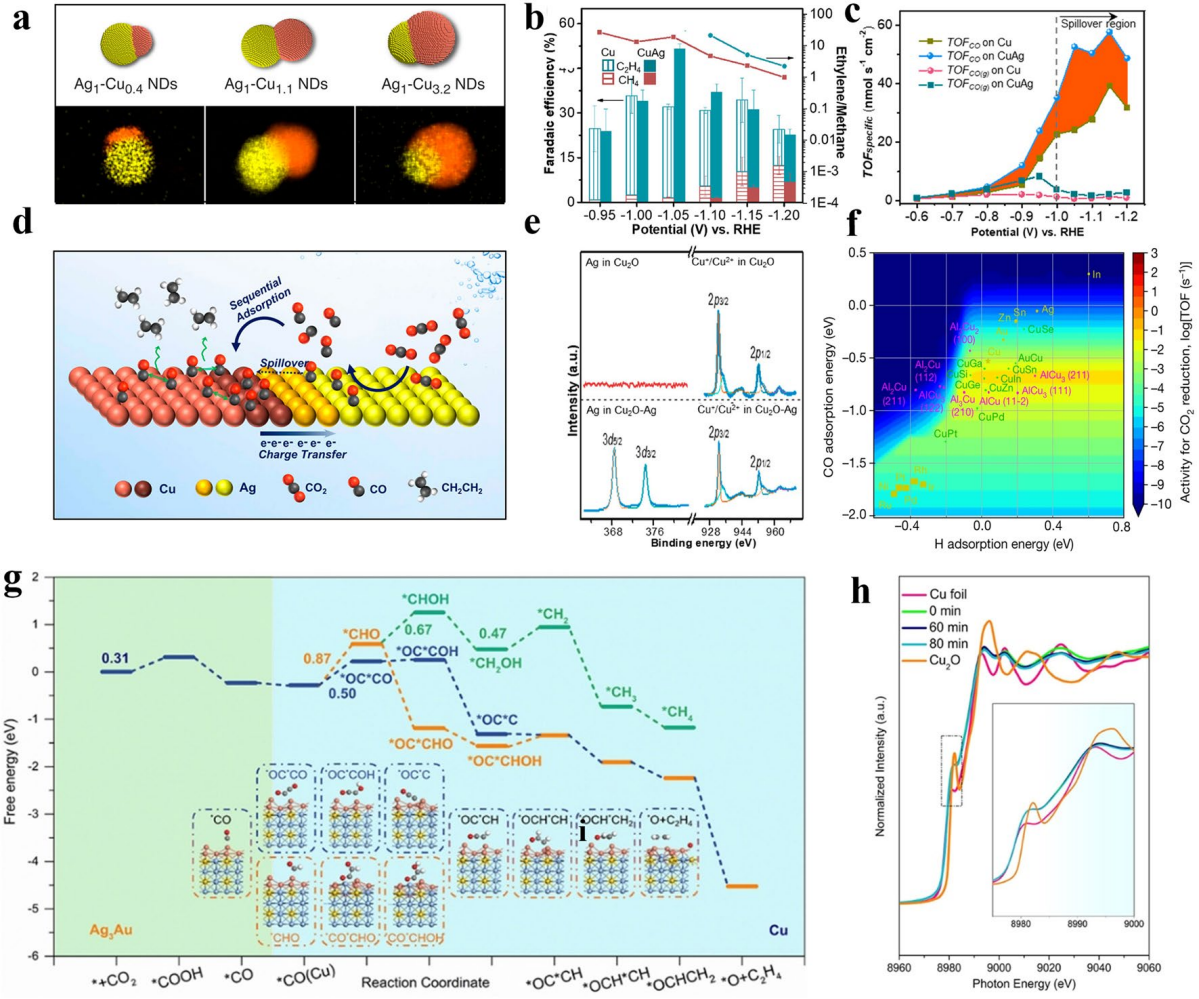
**a** Schematic and EDX elemental mapping of Ag (yellow) and Cu (orange) of Ag_1_-Cu_0.4_ nanodimers, Ag_1_-Cu_1.1_ nanodimers and Ag_1_-Cu_3.2_ nanodimers [159]. Reused with approval; Copyright 2019 American Chemical Society. **b** FE_C2H4_ FE_CH4_ and FE_C2H4_/FE_CH4_ on Cu and CuAg catalysts.** c** The formation rate of CO at the Ag site in CuAg. The orange part indicated that the addition of Ag promotes the formation of CO.** d** Schematic diagram of the proposed mechanism of CO_2_ conversion C_2_H_4_ in the AgCu tandem catalyst. **e** XPS spectra of Cu 2*p* and Ag 3*d* of Cu_2_O-Ag and Cu_2_O [156]. Reused with approval; Copyright 2022 Wiley.** f** 2D activity volcano plot for CO_2_ reduction [30]. Reused with approval; Copyright 2020 Springer Nature.** g** Free energy of intermediates with the reaction coordinates following the route towards CH_4_ and C_2_H_4_.** h** Operando Cu K-edge XAFS spectra of Cu_3_-Ag_3_Au nanoframes at a different time [163]. Reused with approval; Copyright 2021 Wiley

It has been established that the concentration of *CO on catalysts is a decisive factor in determining the selectivity of CO_2_RR to ethylene. Forming alloys by introducing foreign metals (e.g., Ag, Zn, Au, Zr) to Cu can convert CO_2_ to *CO more efficiently, which increases the local *CO concentration around the Cu sites [10, 41, 149–155]. It has been confirmed by operando Roman Spectroscopy that in AgCu alloys, the *CO intermediates initially formed on Ag sites, which were then transferred to the Cu sites for spillover and hydrogenation (Fig. 9b–e) [156]. To confirm this, the nanoporous CuAg catalysts with varied Ag content (3%–9%) have been achieved by the electrodeposition method, and the results indicated that the Ag content influenced the selectivity of CuAg alloys [154]. The low Ag content of 3% decreased the chance to form C_2_H_4_, and the high Ag content of 9% led to the generation of CO by inducing the separated atomic structure [157, 158]. CuAg alloys with appropriate Ag value (6%) resulted in the highest yield of C_2_H_4_ (FE = 60% at − 0.7 V vs. RHE and a total current density of − 300 mA cm^−2^). The regulated CO_2_RR performance by changing the Ag content is attributed to the alteration of the concentration of surface *CO intermediates, which is more conducive to the C–C coupling on Cu sites, which boosted ethylene production. Another example is the AgCu alloy with heterojunction structure which showed the highest yield of C_2_H_4_ with FE = 42% (at − 1.10 V vs. RHE) with Ag content of 6 wt% (Fig. 9a) [159]. The continuous increase of Ag content led to the aggregation of nanoparticles and decrease the catalytic activity. Moreover, the electronic effect induced by the Ag/Cu dual metal alloy catalysts is another factor affecting product selectivity [160]. The results showed that the emergence of the Ag/Cu interface caused electrons to move from the Cu domain to the Ag domain [160]. The electron-depleted Cu could efficiently enhance the binding of *CO on the active sites, which in turn facilitates the coupling of *CO into C_2_H_4_. Owing to the *CO spillover effect and electron transfer effect of tandem catalysis, the selectivity of C_2_H_4_ was further improved in CO_2_RR.

Zn atoms play a similar role to the above-mentioned Ag when alloying with Cu to selectively promote ethylene production. For instance, the mixed CuZn NPs with homogeneous elemental distribution have been derived from the ablation of CuZnO alloy using a nanosecond pulse laser [161]. The uniform distribution of Cu and Zn atoms efficiently reduced the transfer distance of the formed *CO intermediates from Zn sites to the nearby Cu sites, thus reducing the reaction barrier and facilitating the C_2_H_4_ generation. Similarly, incorporating active metal Al into Cu forming Cu-Al alloy can also enhance C_2_H_4_ production (Fig. 9f) [30]. The as-prepared Cu-Al alloy possessed the highest number of *CO adsorption sites, and the *CO adsorption energy (ΔE_CO_) produces near-optimal activity. The water near the Al atoms efficiently reduced the generated *HOCCH to *CCH, which could not only decrease the formation energy, but also inhibits the production of competing alcohols, leading to the selectivity of C_2_H_4_ (FE = 80% at − 1.5 V vs. RHE and at a current density of 400 mA cm^−2^). Different from Zn and Ag to promote the formation of *CO intermediates, Pd possesses stronger adsorption of proton, which can also promote the generation of C_2_H_4_. For instance, phase-separated CuPd alloy with adjacent Cu atoms (phase separation) was beneficial to the production of C_2_H_4_, whereas the one with alternating Cu-Pd arrangement preferred the production of CH_4_ [162]. The adjacent characteristics of Cu atoms allowed favourable molecular distance and small spatial steric hindrance, making the adjacent adsorbed *CO easily dimerize into *COCOH intermediates with the aid of Pd, which were then converted into C_2_H_4_ (FE = 48% at − 0.7 V vs. RHE and the highest total current density of 370 mA cm^–2^). In addition, the ternary Cu-Au/Ag nanoframes could also efficiently promote the reduction of CO_2_ to C_2_H_4_ [163]. Alloyed Ag/Au nanoframes have been recognized as one of the most efficient CO-specific catalysts, and the design of nanopore frameworks is also conducive to the exposure of active centers while promoting mass transfer. The derived Cu-Au/Ag nanoframes have exhibited a maximum value of FE_C2H4_ ≈ 79% at − 0.65 V vs. RHE in the flow cells (Fig. 9g, h). The improved C_2_H_4_ production was ascribed to the integrated tandem catalysis (*CO was formed on the Ag/Au nanoframes and then spill over to Cu for coupling), electronic modulation (Cu conducts electrons to Ag/Au) and defect engineering (Ag/Au framework with many defects).

### Defect Effect

The defects can also play a significant role in the electrocatalysis process, such as optimizing the reaction barrier, increasing the number of active sites, and inhibiting the competitive reaction HER, thus promoting catalytic activity and selectivity [100, 110, 164, 165].

#### Metal Defects

**Fig. 10:**
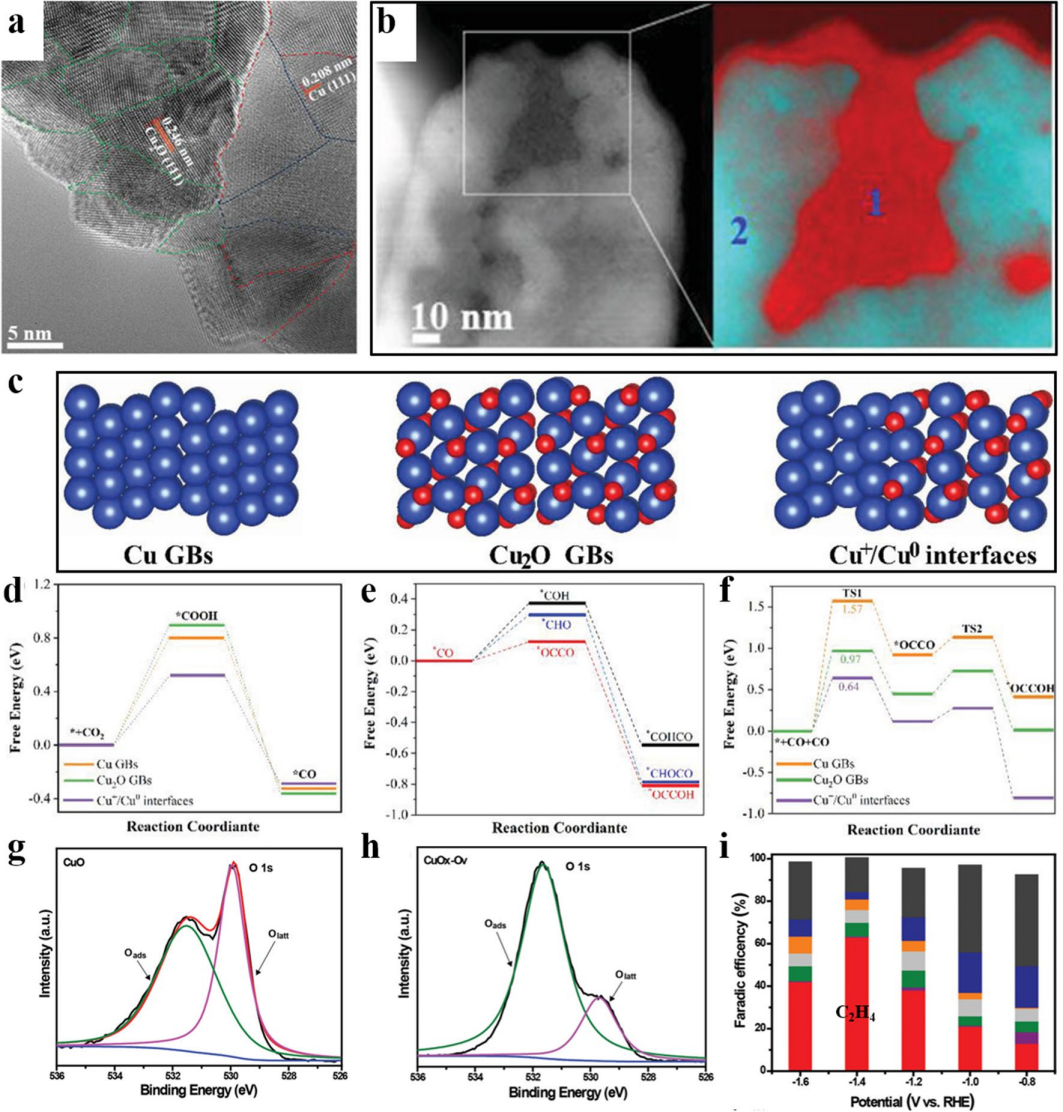
**a** HRTEM image with magnified region of GBs. **b** EELS mapping of Cu^+^ in 1 and Cu^0^ in 2. **c** DFT calculations models for Cu GBs, Cu_2_O GBs, and Cu^+^/Cu^0^ interfaces. The free energy on Cu GBs, Cu_2_O GBs, and Cu^+^/Cu^0^ interfaces of **d** CO_2_ to produce *CO, **e** *CO formate different C_2_ intermediates, **f** *CO dimerization to obtain *OCCO and subsequent *OCCO conversion to *OCCOH [168]. Reused with approval; Copyright 2022 Wiley. XPS spectra of O 1s of **g** CuO and **h** CuO_x_-Vo. **i** FE_C2H4_ of CuO_x_-Vo [169]. Reused with approval; Copyright 2019 Wiley

Metal defects optimize the CO_2_ conversion to C_2_H_4_ by altering unsaturated coordination atoms, electronic band structure and local charge distribution. For example, the prism-shaped Cu catalysts with metal defects increased C_2_H_4_ yield due to the possible change of local pH and the formation of low-coordinated Cu atoms on the rough prism surface [166]. Cu nanosheets with 6 nm defects size also enhanced the FE_C2H4_ to 83% at − 1.2 V vs. RHE [25]. The high performance was ascribed to the induced defects, which are beneficial to the adsorption, enrichment and restriction of reaction intermediates (*CO and *OCCO) and OH^−^, thus promoting the C–C coupling for ethylene production. The effect of pore defects on the performance of C_2_H_4_ generation has also been studied. Changing the pore size and depth can significantly influence the selectivity of designed catalysts. When the pore width was reduced to 30 nm, the formation of C_2_H_4_ increased from 8% to 38%, while the C_1_ gas products decreased (FE_CO_ = 5% and FE_CH4_ = 15%) [167]. The enhanced C_2_H_4_ selectivity is attributed to the restriction of proton transmission and the increased local pH, which is conducive to the occurrence of C–C coupling. Moreover, grain boundaries have been investigated as a kind of defect by designing a unique planetary-like Cu structure via structural reconstruction (Fig. 10a, b) [168]. A large number of Cu grain boundaries were produced during the reconstruction process due to the fusion of components in the ultrafine Cu particles. The components in the shell were separated to form a nano-gap structure, which could effectively limit OH^−^ on the surface of catalysts to maintain a high local pH value, thus promoting C_2_H_4_ production (Fig. 10c–f).

#### Vacancies Defects

In addition to metal defects, oxygen vacancies have also been investigated to selectively boost C_2_H_4_ production. These oxygen vacancies can not only generate unsaturated metal ions, but also induce the charged positions as trap sites for electrons or holes [45]. For instance, highly branched CuO_x_ nanodendrites with a high surface density of Vo's (referred to as CuO_x_-Vo) prepared by a two-step thermal annealing and electrochemical reduction technique showed a high FE_C2H4_ of about 63% at − 1.4 V vs. RHE (Fig. 10g, h) [169]. This Vo's possessed weakly bound electrons, which make them excellent Lewis base sites to adsorb CO_2_ and transfer electrons to the intermediate of CO_2_^·−^. Therefore, Vo's is functional to optimize the adsorption energy of reactants and promote molecular activation, leading to the strong adsorption energy of *CO/*COH intermediates and rapid desorption of *CH_2_ (2*CH_2_ → C_2_H_4_).

In addition to O vacancies, other anionic defects such as S and N vacancies have also been found to have an impact on CO_2_ reduction products. Due to the high mobility of S atoms in complex CuS crystal structures, it is easy to produce stable surface defects [170, 171]. For example, a copper sulfide-copper core-shell catalyst rich in surface vacancies (Cu_2_S-Cu) was synthesized by using enriching vacancy Cu_2_S nanoparticles as precursors, which can achieve high selectivity for C_2_H_4_ (45% at − 1.1 V vs. RHE) [172]. The introduced vacancies on the Cu shell can efficiently modify the electronic structure of adjacent Cu atoms, which can affect the energy barrier of the rate-limiting reaction intermediates. It is worth noting that when the proportion of vacancies on the surface increases, the energy barrier for ethylene formation will be slightly increased, but ethanol will not be affected, so the selectivity of the product will be transferred from C_2_H_4_ to multi-carbon alcohols. Therefore, optimized vacancy degree can contribute to the ethylene production [173]. Recent studies have also shown that N vacancies possess higher stability than O vacancies due to the high metal-N bond energy in transition metal nitrides [174, 175]. Cu_3_N_x_ with different nitrogen densities were prepared by a lithiation-enabled vacancy-engineering strategy, and the nitrogen vacancy densities highly affect the adsorption energy of *CO and the energy barrier for the formation of key C_2_ intermediates [176]. These presented N vacancies led to a decreased distance between adjacent Cu atoms, which is beneficial to the bonding of *CO absorbed at Cu sites [177]. The FE of Cu_3_N_x_ with 50% of N vacancy concentrations for C_2_ products is 81.7% (FE_C2H4_ = 56%) at − 1.15 V vs. RHE [176]. The Cu_3_N_x_ catalyst also showed excellent electrochemical stability at high current density (*j* = − 350 mA cm^−2^, 10,000 s, FE_C2_ dropped from 82.1% to 76%), which greatly surpasses the CuO_x_ catalyst with oxygen vacancies (*j* = − 350mA cm^−2^, 850 s, FE_C2_ dropped from 63% to 43%).

#### Non-metal Doping

Incorporating non-metal is another technique to introduce defects in Cu-based catalysts. For instance, Cu_3_N nanocube with perovskite structure preferred the coupling of *CO and *CHO thus enhancing the C_2_H_4_ production [178]. Cu_1.8_Se nanowires grown on 3D copper foam substrates also exhibited excellent capability to produce ethylene, reaching the maximum value of FE_C2H4_ of 55% at − 1.1 V vs. RHE [179]. The results showed that the introduction of Se can reduce the surface concentration of *CO and inhibit the formation of C_1_ products. In addition, fluorine-modified copper (F–Cu) introduced a new view on the mechanism of "hydrogen-assisted C–C coupling" [39]. Fluorine modification not only is conducive to the activation of H_2_O to form active hydrogen species (*H), but also promotes the adsorption and hydrogenation of *CO to produce *CHO intermediates, which can be easily coupled and reduced to C_2+_ products on the Cu surface. Other than single non-metal elements, silicon dioxide has also been introduced into Cu by one-pot coprecipitation method to create active Cu-SiO_x_ interface sites, which reduced the formation energy of key intermediates OCOH* and OCCOH* during ethylene production [180].

### Surface Modification

**Fig. 11:**
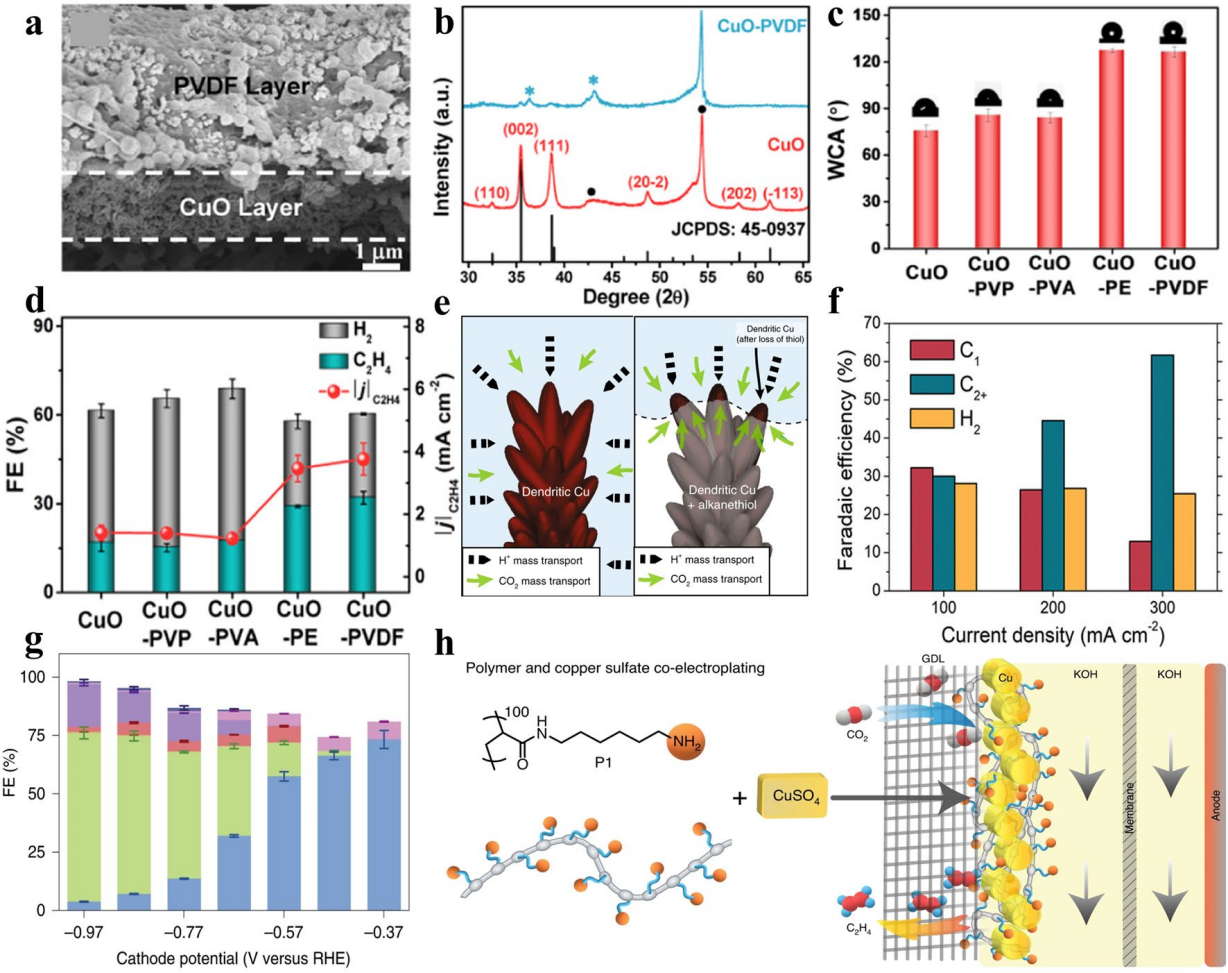
**a** Cross-sectional morphology in SEM pictures. **b** XRD pattern compared with standard CuO-PVDF, where “*” denotes diffraction peaks from PVDF and “•” from graphite [182]. Reused with approval; Copyright 2021 American Chemical Society. **c** Water contact angles,** d** FE_C2H4_, FE_H2_ and *j*_C2H4_ for CuO surface modified with PVP, PVA, PE, and PVDF [185]. Reused with approval; Copyright 2019 Wiley. **e** Mass transfer diagram of non-hydrophobic and hydrophobic dendrites [32]. Reused with approval; Copyright 2019 Springer Nature. **f** FE of C_1_, C_2+_ products and H_2_ at 3 kinds of current densities [34]. Reused with approval; Copyright 2019 Wiley. **g** FE of all products on Cu-P1 at the range of − 0.37 V to − 0.97V vs. RHE. **h** Schematic illustration of synthetic Cu-P1 on the GDL [186]. Reused with approval; Copyright 2021 Springer Nature

Changing the hydrophobicity at the interface between catalysts and electrolyte, such as incorporating molecules to modify the surface of catalysts, is an efficient approach to inhibit HER, facilitate the mass transfer of CO_2_, and optimize the adsorption/desorption of intermediates, thus boosting the catalytic activity and selectivity of CO_2_RR to ethylene. Moreover, increasing pH at the hydrophobic interface is also conducive to the occurrence of C–C coupling [181]. For instance, coating different hydrophilic/hydrophobic polymers on CuO nanoparticle (NPs) electrodes can efficiently alter the electrode-electrolyte interface in CO_2_RR process (Fig. 11a, b) [182]. It was demonstrated that hydrophilic polymers played negligible roles in altering the catalytic performance, whereas hydrophobic polymers can significantly improve the activity, selectivity and stability of CuO derivative electrodes to boost C_2_H_4_ generation (Fig. 11d). The experimental results demonstrated that the enhanced performance was mainly attributed to the constructed hydrophobic microenvironment of the gas/liquid/solid three-phase interface on the electrode surface, which limited the water diffusion, improved the local pH near the electrode surface, and then improved the performance of CO_2_RR process (Fig. 11c, e) [32, 183, 184]. The incorporated polymers can also work as the protective layer of the catalysts. For example, introducing N’N-ethylene-phenanthrolinium dibromide in Cu catalysts can efficiently etch the surface to form cubic nanostructures and stabilize the nanostructures in the electrocatalysis process by forming a protective organic layer, resulting in a high selectivity of 45% for C_2_H_4_ at − 1.07V vs. RHE [185]. Moreover, a molecular regulation strategy by functionalizing the electrocatalysts with organic molecules can stabilize the intermediates (*CO) and promote the conversion of CO_2_RR to ethylene [14]. The incorporation of organic polymers with specific functional groups has been investigated to tune the selectivity of the CO_2_RR process. The Cu-poly-N-(6-aminohexyl)acrylamide (denoted as Cu-P1) prepared by the coprecipitation method boosted the ethylene selectivity up to 72% at − 0.97 V vs. RHE in 1 M KOH (Fig. 11g) [186]. A series of stable Cu electrodes modified by amine-containing additives with different methylation degrees were prepared by controlling the amount of methyl to replace the hydrogen atom on P1. The results demonstrated that the CO_2_RR selectivity was dependent on the degree of methylation of P1 on the Cu-P1 electrode, in which the ethylene yield decreased with the increase in the degree of methylation in the polymer, whereas the HER process was the opposite trend. Amino groups also play a key role in boosting the C_2_H_4_ generation by contributing to the CO_2_ capture and leading to local pH increase on the electrode surface (Fig. 11h). Ionic liquids (IL) coated on the Cu catalysts are responsible for the improved conversion efficiency of CO_2_ to ethylene, which is attributed to their strong CO_2_ solubility, effectively increasing the CO_2_ concentration in the reaction phase and improving the equilibrium conversion rate. On the other hand, the strong hydrogen bond and static electricity between ionic liquids and CO_2_ show suitable reaction energy, which can partially activate CO_2_ double bond and achieve stronger adsorption of *CO intermediates [187, 188]. For instance, IL BmimNO_3_ (IL 1-butyl-3-methylimidazolium nitrate) on Cu electrocatalyst delivered FE = 77.3% for ethylene selectivity at − 1.49 V vs. RHE [189]. The high CO_2_RR performance was ascribed to the activation of CO_2_ molecules on IL, the changed coordination environment of Cu atoms, regulated local electronic structure, and the boosted *CO dimerization rate. Moreover, the hydrophobic carbon chain in BmiNO_3_ increased the CO_2_ concentration at the electrode-solution interface, thus inhibiting the competition of HER and improving the efficiency of CO_2_RR.

### Valence State

The valence state of metal atoms also plays an important role in the selectivity of CO_2_RR [190]. When the surface Cu atoms are partially positively charged, the synergistic between Cu^δ+^ and Cu^0^ causes strong electrostatic adsorption of the negatively charged *CO intermediates on the active sites, reducing the C–C coupling energy barrier and promoting ethylene formation [191]. For instance, Cu_2_O with Cu^+^ was covered by the reaction intermediates (such as *CO) on the surface in the CO_2_RR process, which effectively prevents Cu^+^ from being reduced to Cu [192]. Cu^+^ has lower adsorption energy of *CO intermediate than Cu, so it can improve the coverage of *CO and promote C–C coupling. The achieved catalyst reached a maximum C_2+_ FE of 75.2% at a potential of − 0.61 V vs. RHE. Moreover, the catalysts with a mixed valence state may exhibit superior performance compared to the ones with a single valence state [193]. In the Cu^0^–Cu^+^ system, the Cu^0^ functioned to activate CO_2_ and promote the subsequent electron transfer, meanwhile Cu^+^ site enhanced the adsorption of *CO, thus further promoting C–C coupling [194–197]. The selectivity of the catalysts with Cu^+^/Cu interface to C_2_H_4_ can reach 40% at − 1.0 V vs. RHE, while very little C_2_H_4_ (less than 5%) has been observed on pristine Cu catalysts. In addition, Cu_4_O_3_ with the same amount of Cu^+^ and Cu^2+^ ions were prepared by the solvothermal method, which acted as pre-catalysts to form the intrinsic active sites (Cu^0^, Cu^+^ and Cu^2+^) after partial reduction [34]. The active sites with mixed valence states can not only reduce the energy barrier in forming C–C bonds, but also possess the capability to electrostatically stabilize *OCCO intermediate. The FE_C2H4_ was 43% at − 0.64 V vs. RHE, and the highest C_2+_/C_1_ product ratio of 4.8 has been achieved at the same condition (Fig. 11f). Table 1 summarizes the performance of CO_2_ to ethylene for the Cu-based catalysts, including FE, current density, durability, electrochemical cell type, GDE structure, and electrolyte selection.

**Table 1:** A summary of recent advances in CO_2_ electroreduction to C_2_H_4_

Strategy	Catalyst	Electrolyte	FE_C2H4_ (%)	E (V vs RHE)	j_C2H4_ (mA cm^-2^)	GDE structure	electrochemical cell	durability (h)	Refs.
Nanostructure effect	Cu-I	0.1 M KHCO_3_	80	− 0.9	31.2	–	H-cell	22	[198]
	OBC	0.5 M KHCO_3_	45	− 0.95	44.7	–	H-cell	10	[199]
	4H Au@Cu nanoribbon	0.1 M KHCO_3_	44.9	− 1.11	14.4	–	H-cell	–	[28]
	KB@Cu_3_ (HITP)_2_	0.1 M KHCO_3_	70	− 1.37	26.5	–	H-cell	10	[200]
	Cu-mesocrystal	0.1 M KHCO_3_	27.2	− 0.99	6.8	–	H-cell	6	[134]
	CuAl-1	0.1 M KHCO_3_	82.40	− 0.99	–		H-cell	100	[201]
	Cu-N-C-800	0.1 M KHCO_3_	24.8	− 1.4	6.84	–	H-cell	10	[202]
	DVL-Cu	0.5 M KCl	84.5	− 0.8	92.5	–	MEA	55	[122]
Molecular catalysis	Cu-12	1 M KHCO_3_	72	− 0.83	232	PTFE	Flow-cell	190	[14]
	PcCu-Cu-O	0.1 M KHCO_3_	50	− 1.2	7.3	–	H-cell	4	[147]
	Ag@BIF-104NSs (Cu)	0.5 M KHCO_3_ + 0.5 M KCl	21.43	− 1.2	4.36	–	H-cell	4	[203]
	S-HKUST-1	1 M KHCO_3_	60.0	− 1.30	20	–	H-cell	8	[146]
	CTPI	0.1 M KHCO_3_	66	− 3.7	208	PTFE	Flow-cell	100	[204]
	A-CuNW	0.1 M KHCO_3_	77.4	− 1.01	22.4	–	H-cell	200	[27]
	IL@Cu	0.1 M KHCO_3_	77.3	− 1.49	34.2	–	H-cell	3	[189]
	C/HKUST-1/Cu/PTFE	1 M KOH	49.1	–	491	PTFE	Flow-cell	65	[37]
	Cu-PzH	1 M KOH	60	− 1.0	346	CP	Flow cell	3.9	[205]
	GMC-[Cu_2_(NTB)_2_]	0.1 M KCl	42	− 1.28	–		H-cell	2	[206]
	AuNN@PCN-222(Cu)	0.1 M KHCO_3_	52.5	− 1.2	–	–	H-cell	10	[207]
Alloy effect	Cu-Al	1 M KOH	80	− 1.5	400	PTFE	Flow-cell	50	[30]
	Cu/NiNC	1 M KOH	40	3.2	150	–	MEA	–	[208]
Defect effect	Cu_3_N	0.1 M KHCO_3_	60	− 1.6	30	CP	Flow-cell	20	[178]
	F-Cu	2.5 M KOH	60	− 0.54	800	CP	Flow-cell	40	[39]
	Cu_2_S/Cu-V	1 M KHCO_3_	21.1	− 0.95	–	–	H-cell	–	[172]
Surface modification	C/Cu/PTFE	0.5 M KHCO_3_ + 0.5 M KCl	70	− 0.89	350	PTFE	Flow-cell	50	[109]
	Hydrophobic Cu dendrite	0.1 M CsHCO_3_	56	-	–	–	H-cell	–	[32]
	Cu-KOH	1 M KOH	54.5	3.25	153	–	MEA	6	[209]
	Cu:Py:SSC	3 M KOH	65	2.6	150	PTFE	MEA	110	[210]
	MgAl-LDH/Cu	1 M KHCO_3_	55.1	–	–	CP	Flow-cell	7	[211]
Valence state	Cu(B)-2	0.1 M KCl	53±1	− 1.0	55	–	H-cell	40	[212]
	AN-Cu	1 M KHCO_3_	38.1	− 1.08	7.3	–	H-cell	40	[213]
	Oxide-derived Cu	0.1 M KHCO_3_	60	− 0.9	–	–	H-cell	–	[214]
	P_0.075_-Cu	0.1 M KHCO_3_	30.7±0.9	− 1.6	17.57	–	H-cell	25	[215]
	Cu-CuI	1 M KOH	71	− 0.87	276	–	Flow-cell	85	[216]
	Cu-SiO_x_-2.5	0.1 M KHCO_3_	65	− 4.1	215	PTFE	MEA	50	[180]

## Conclusions and Perspectives

The rational design of Cu-based electrocatalysts for ethylene production with high energy density and selectivity is one of the most significant goals in CO_2_RR. In this review, we have summarized the key steps to convert CO_2_ to ethylene, including CO_2_ adsorption/activation, formation of *CO intermediate, and the C–C coupling step. An in-depth understanding of these key steps provides an intrinsic mechanistic investigation of fundamental principles for the CO_2_RR process. The microenvironment (pH effect, cation/anion effect), facet, *CO intermediates, overpotential and additives influence on CO_2_RR to generate ethylene or other competitive products have been discussed to propose the preferred reaction pathways and conditions to selectively produce ethylene. The catalyst preparation and surface engineering at the atomic level can significantly affect the interface microenvironment, thus the selectivity from CO_2_ to ethylene. The strategies of nanostructures control, molecular catalysis, alloy, defect engineering, surface modification, and oxidation state alteration to engineer the Cu-based catalysts have been summarized and discussed. The summarized recent progress of research work demonstrated that the engineered strategies are highly associated with the binding energies of intermediates (optimize the binding energy for specific intermediates), thus controlling the catalytic activity, selectivity and product distribution of CO_2_RR.

Currently, tremendous progress has been achieved for CO_2_RR based on experimental and computational methodologies, resulting in many breakthroughs for CO_2_ converting to ethylene or other C_2+_ product. However, it is still challenging to specifically generate single specific products as it is difficult to control the engineering strategies to have no influence on other intermediates or products. Therefore, it is still necessary to address the fundamental issues related to CO_2_RR to achieve continued development and practical application.

### Full Mechanistic Picture

The multiple electrons/protons transfer and a large number of reaction intermediates make CO_2_RR process extremely complicated [17]. Moreover, the catalytic activity and selectivity of CO_2_RR are related to many parameters, such as the catalyst structure and composition (facet, defects, etc.) and reaction conditions (pH, applied potential, electrolyte, etc.), making it even more difficult to achieve the desired products due to the competitions between different reaction pathways. Moreover, the competing HER also decreases the total production of ethylene. It is difficult to suppress H_2_ evolution since *H is necessary for CO_2_RR mechanism. The selective effect of secondary metals with different binding strengths for *H and *O is vital to alter the products [217]. When secondary metals with weak *H and *O binding energy (such as Ag, Au) form bimetallic catalysts with Cu, due to the synergistic influence of the electronic and geometric effects of the alloy, the weak adsorption energy to *CO is caused, which is facilitates C–C coupling [218]. And weak *H binding also inhibits HER and improves the overall CO_2_ utilization rate. Therefore, selecting a secondary metal with an appropriate binding energy of the intermediates can promote the production of ethylene.

### Stability Issues

In addition to the catalytic activity and selectivity for CO_2_RR to generate ethylene products, stability is also important to evaluate the catalytic capability of CO_2_RR electrocatalysts [219]. Currently, most catalysts can only last less than a few hundred hours under operation conditions. This is far from satisfactory in terms of the practical applications of CO_2_RR technologies. Most reports focus on the current density and product selectivity, whereas the stability of catalysts has not received enough attention. Finding a key point to balance the relationship between catalytic activity and stability is desired. Moreover, the degradation mechanisms and corresponding solutions for enhancing the stability of catalysts are needed. Wrapping or embedding Cu-based catalysts in ultrathin layers or anchoring Cu-based materials on stable substrates may achieve excellent stability with no sacrifice for the catalytic activity.

### Operando/In-Situ Techniques and DFT Calculation

Operando/in-situ techniques and DFT calculation are effective tools for understanding the structure-performance relationship [220]. Structure evolution of the catalysts may occur in the CO_2_RR conditions, and the operando/in-situ characterization techniques could monitor the dynamic process to identify the real active sites. Moreover, the intermediates can also be detected in real-time for specific reaction pathways. DFT calculation is a powerful tool for acquiring the reaction-free energy of CO_2_RR for specific products, revealing the adsorption energies of intermediates and providing an in-depth understanding of the catalytic mechanism at the atomic level. Future efforts may pave the direction of enhancing the time and spatial resolution of operando characterization, and conducting machine-learning coupled DFT to in-depth explore the catalytic mechanism and predict the efficient electrocatalysts.

### Designing Advanced Electrolyzers

Other than the designed catalysts and mechanism understanding of CO_2_RR, electrolyzers are also important in practical applications [221–224]. Currently, H-cells are the most widely used devices for CO_2_RR, owing to their low cost and simple setup [50]. However, the limited solubility and diffusion of CO_2_ in conventional H-cell electrolytes lead to low current densities (< 100 mA cm^−2^) which limits their commercialization [5, 55, 225]. Therefore, H-cells are usually used in the laboratory for preliminary performance tests. Flow cell is another conventional electrolysis cell. Different from H-cell, CO_2_ flows continuously from the back to the catalyst surface through the gas diffusion layer (GDL). Within the flow cell, the rich three-phase boundary composed of CO_2_, catalyst and electrolyte significantly improve the reaction efficiency [50, 59, 135]. The advantages of a short CO_2_ diffusion path and no solubility limitation strengthen the mass transfer of CO_2_, thereby improving the current density and selectivity [59]. However, conventional hydrophobic carbon-based GDL faces the problem of being flooded after a few hours of testing, resulting in flow cell failure. Modification using porous polytetrafluoroethylene (PTFE) as GDL can greatly improve the hydrophobicity and stability of flow cells [29, 226]. However, it is still severe that the reaction of CO_2_ with alkaline electrolyte to form carbonate will also lead to low single-pass utilization of CO_2_ and the reduction of local pH [55]. Membrane electrode assemblies (MEAs) remove the catholyte and allow the direct contact between the cathode GDL with the exchange membrane, thereby reducing the resistance of the entire cell. Compared to flow cell, MEAs show higher activity and lower energy consumption [14]. However, they still face problems such as low ionic conductivity and low CO_2_ utilization (< 10%). Therefore, additional efforts should be made to improve the ionic conductivity and assembly process of ion exchange membranes in the future [55].
